# Investigating the Link between Molecular Subtypes of Glioblastoma, Epithelial-Mesenchymal Transition, and CD133 Cell Surface Protein

**DOI:** 10.1371/journal.pone.0064169

**Published:** 2013-05-29

**Authors:** Hadi Zarkoob, Joseph H. Taube, Sheila K. Singh, Sendurai A. Mani, Mohammad Kohandel

**Affiliations:** 1 Department of Applied Mathematics, University of Waterloo, Waterloo, Ontario, Canada; 2 Department of Translational Molecular Pathology, University of Texas, MD Anderson Cancer Center, Houston, Texas, United States of America; 3 Metastasis Research Center, University of Texas, MD Anderson Cancer Center, Houston, Texas, United States of America; 4 McMaster Stem Cell and Cancer Research Institute, Michael DeGroote Centre for Learning and Discovery, Hamilton, Ontario, Canada; 5 Centre for Mathematical Medicine, Fields Institute, Toronto, Ontario, Canada; 6 Department of Surgery, McMaster University, Hamilton, Ontario, Canada; NIH/NCI, United States of America

## Abstract

In this manuscript, we use genetic data to provide a three-faceted analysis on the links between molecular subclasses of glioblastoma, epithelial-to-mesenchymal transition (EMT) and CD133 cell surface protein. The contribution of this paper is three-fold: First, we use a newly identified signature for epithelial-to-mesenchymal transition in human mammary epithelial cells, and demonstrate that genes in this signature have significant overlap with genes differentially expressed in all known GBM subtypes. However, the overlap between genes up regulated in the mesenchymal subtype of GBM and in the EMT signature was more significant than other GBM subtypes. Second, we provide evidence that there is a negative correlation between the genetic signature of EMT and that of CD133 cell surface protein, a putative marker for neural stem cells. Third, we study the correlation between GBM molecular subtypes and the genetic signature of CD133 cell surface protein. We demonstrate that the mesenchymal and neural subtypes of GBM have the strongest correlations with the CD133 genetic signature. While the mesenchymal subtype of GBM displays similarity with the signatures of both EMT and CD133, it also exhibits some differences with each of these signatures that are partly due to the fact that the signatures of EMT and CD133 are inversely related to each other. Taken together these data shed light on the role of the mesenchymal transition and neural stem cells, and their mutual interaction, in molecular subtypes of glioblastoma multiforme.

## Introduction

Glioblastoma multiforme (GBM) is currently the most commonly diagnosed and aggressive class of brain tumor. Despite significant advances in chemotherapy, radiotherapy and surgical treatment, the median adult patient survival time, following the diagnosis of GBM, is only 6–12 months [1], [2]. In order to better understand the molecular determinants involved in the development, progression, aggressiveness as well as shortcomings associated with conventional treatments of GBMs, there has been significant increase in research focusing on high dimensional profiling studies of the disease [3], [4], [5], [6]. In particular, genetic profiling has been used to classify glioblastomas into distinct molecular subtypes, and to characterize the key molecular pathways within each subtype. An initial classification scheme separated high-grade gliomas into pro-neural, proliferative and mesenchymal subtypes, exhibiting either neuronal or neural stem cell markers [5]. More recently, Verhaak *et al*. [7], using data obtained from The Cancer Genome Atlas (TCGA) [8], re-classified GBMs into four genetic subtypes (mesenchymal, classical, neural and pro-neural) characterized by aberrations in genes including PDGFRA/IDH1, EGFR, and NF1, among others.

Induction of EMT leads to an increased potential for cell migration, changes in cytoskeletal organization and reduced cellular adhesion and has been shown to be a mechanism leading to the metastatic invasiveness of many carcinomas [9]. Recent studies have also linked EMTs with the acquisition of stem-cell-like characteristics [10], [11]. One hurdle in studying the role of EMT in pathological studies has been the lack of a comprehensive characterization of genetic changes occurring in this process. Recently, Taube *et al*. [12] studied the characterization of gene expression signatures in human mammary epithelial cells induced to undergo an EMT, by expressing Gsc, Snail, Twist, TGF-β1 and knocking down E-cadherin, and were able to propose a robust signature for EMT from overlapping changes in gene expression patterns. In this paper, we use this EMT-signature to study the mesenchymal transition of GBMs.

While the cell(s) responsible for initiating GBMs have not yet been definitively identified, recent studies have reinforced the hypothesis that human gliomas may have a neural stem cell lineage [13], [14], [15]. The cell surface protein CD133 has been used to extract a subset of putative stem cells in GBM. Experimental evidence demonstrates that uncultured CD133+ cells have a higher frequency of tumor initiation in mice [16], and furthermore that these cells display features of radioresistance and chemoresistance [17], [18]. More recent studies demonstrate that CD133^+^ cells resemble the genotype of human embryonic and neural stem cells, and that they can be used to identify an aggressive subpopulation of GBM [19]. In spite of this evidence for the usefulness of CD133 as a marker for tumor initiating cells, there are studies that report on the development of CD133+ cells from fresh CD133− cells [20]. Further studies are required to highlight the origin of GBM cells [21], [22].

Studying the link between GBM molecular subtypes and the EMT process, and the connections of these with the putative stem cell origin of GBMs can play an important role in understanding the mechanisms associated with the development and aggressiveness of GBM [23]. Here using genetic data from TCGA, we study the role of EMT in each of the GBM molecular subtypes. We also use molecular profiling to identify a genetic signature for the CD133 cell surface protein, and apply this signature to study the link between CD133 and both the EMT process and the GBM subtypes. Using this data, we are able to shed light on the role of EMT and a stem cell genotype in GBM molecular subtypes.

## Results

### Correlations between Gene Expression Profiles in TCGA GBM and EMT Samples

In order to identify a clear correlation between the development of GBM and the role of EMT in this process, we first examine whether the regulation of key genes involved in the EMT process is evident in the GBM samples. We compare the recently identified core EMT signature [12] with the gene expression signatures of GBM samples obtained from TCGA. At the time when we conducted this study, the TCGA data included 373 GBM samples and 10 normal samples. We identified a genetic signature for GBMs by extracting genes that exhibited at least a two-fold difference in their expression pattern in GBM samples as compared with normal samples (File S1).

We then compared the core EMT genetic signature and the signature obtained from the TCGA samples. We observed a significant overlap between genes that were up regulated in GBMs and those that were up regulated in EMT (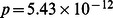, two-sided Fisher exact test (TSFET)). In addition, we observed a significant overlap between genes that were down regulated in GBMs and those that were down regulated in EMT (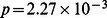, TSFET). Moreover, we noted that there is a negatively significant overlap between genes down regulated in EMT and those up regulated in GBM samples. That is, the overlap between the gene lists was significantly lower than the expected overlap due to pure chance, taking into con sideration the length of the gene lists (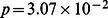; TSFET). We did not observe any significant relation between genes that were up regulated in EMT and down regulated in GBM samples (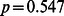; TSFET). We made the comparisons based on the genes whose expression levels were provided in the datasets taken from both TCGA and [12]. These genes formed the background set in the TSFET (see Methods). The number of such genes was 11296. Table 1 provides the number of genes in the EMT and GBM signatures as well as the length of overlap between them. It also provides length of the overlap that we expect due to chance, and the p-value corresponding to the significance of the observed overlaps. Tables S1A and S1B list, respectively, up and down regulated genes in the EMT signature used in this study. Tables S2A and S2B provides genes that are, respectively, up and down regulated in the signatures of both EMT and GBM.

**Table 1:** Comparison of EMT and GBM signatures.

	Overlap (Exp. Overlap)	p-value
**EMT^up^(78)-GBM^up^(1386)**	34 (9.57)	4.53×10^−12^
**EMT^down^(129)-GBM^down^(981)**	22 (11.20)	2.27×10^−3^
**EMT^up^(78)-GBM^down^(981)**	8 (6.77)	5.47×10^−1^
**EMT^down^(129)-GBM^up^(1386)**	8 (15.83)	3.07×10^−2^

Two-sided Fisher exact test (TSFET) is used to compare EMT and GBM signatures. The background set in TSFET includes genes whose expression levels are provided in the data sets taken from both TCGA and (12). The number of these genes in the background set is 11296. The number of genes in the EMT and GBM signatures is provided within the parentheses in the first column of the table. The second column provides length of the overlap between the signatures as well as length of the overlap that we expect due to chance. The third column provides p-values that assess significance of the overlap between the signatures. The first two rows denote cases where the overlap between the signatures is significantly larger than what we expect to pure chance. The last row denotes the case where the overlap between the signatures is significantly less than what we expect to pure chance. Finally, the third row represents the case where the overlap between the signatures is not statistically significant (p-value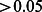).

### Molecular Clustering of TCGA GBM Data and of EMT Expression Signature among GBM Subtypes

Recently, the neural, proneural, classical and mesenchymal GBM subtypes were identified and characterized using data obtained from TCGA [7]. We aimed to see whether the differential expression of EMT related genes varies among these GBM molecular subtypes. We were particularly interested in the mesenchymal subtype because it has previously been shown to express a number of EMT related genes [5], [7], [20]. Verhaak *et al*. [7] clustered TCGA data from 200 GBM samples and 2 normal brain samples. When the studies, described here, were conducted, the TCGA dataset had grown to 373 GBM and 10 normal brain samples. Thus, we sought to first determine whether the same clusters obtained in the original study were obtained when the updated TCGA dataset was used.

#### Re-clustering of TCGA data

**Figure 1:**
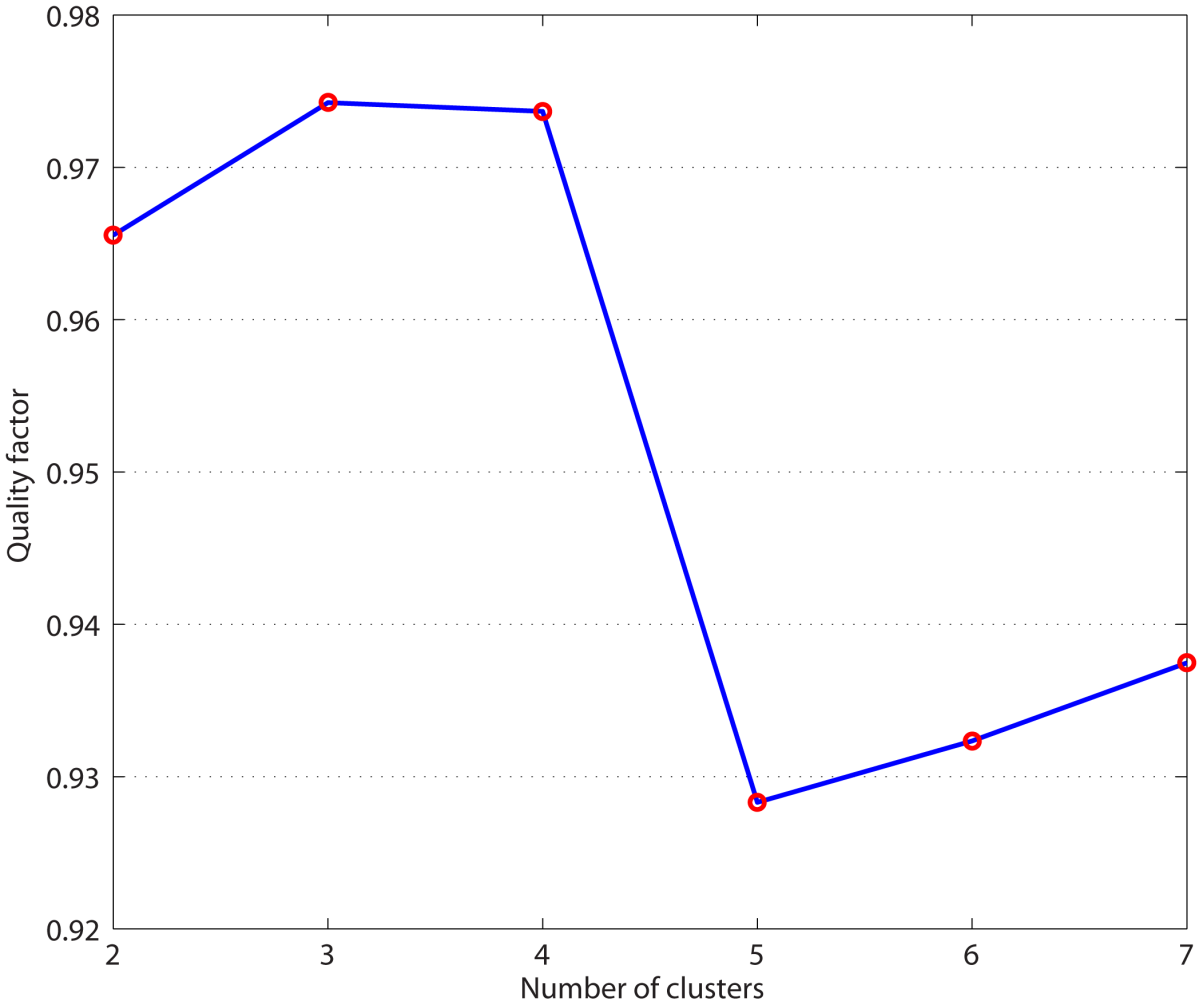
Quality factor as a function of number of clusters.

**Figure 2:**
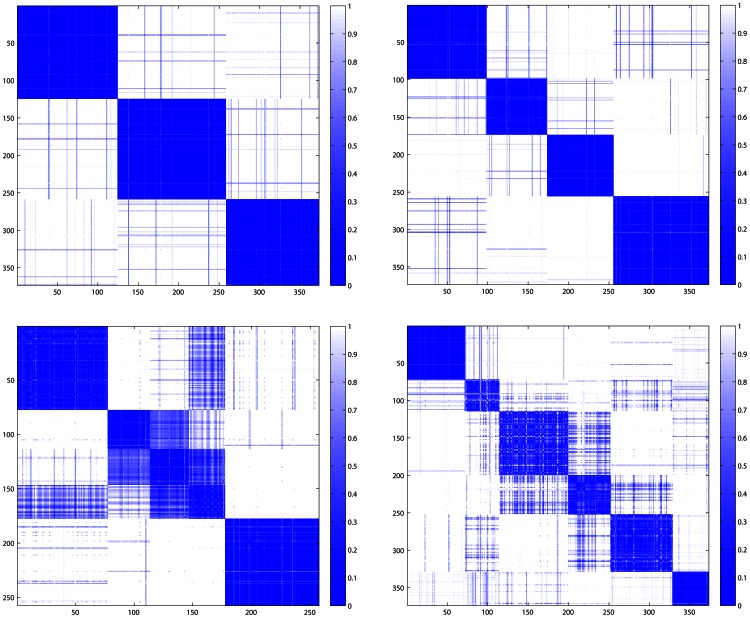
Consensus matrices for the number of clusters k = 3 (top left), 4 (top right), 5 (bottom left) and 6 (bottom right). Numbers on the horizontal and vertical axes represent index of the samples.

We used level 3 gene expression data of 373 GBM and 10 normal samples obtained from Affymetrix gene chips. Before performing the re-clustering of the TCGA GBM data, we performed a filtering step to include only those genes with median absolute deviation (MAD) of at least 0.5 across 373 GBM samples. We then normalized the remaining data such that each row (representing a gene) has zero mean and unit variance. While the original study [7] used consensus clustering [24] in association with average linkage hierarchical clustering, we used consensus clustering in association with k-means clustering which results in more robust clusters (Figure S1 and Figures 1 and 2). We measured the quality of clusters using a quality factor that increases as the consensus matrix became cleaner, that is, its elements become closer to 0 or 1 (see Methods). The quality factor for cluster numbers k = 2 to k = 7 is presented in Figure 1. It can be seen that the quality factor has a sharp drop from k = 4 to k = 5 clusters that supports the existence of four clusters in the GBM samples. The consensus matrices corresponding to k = 2, 3, 4 and 5 matrices are presented in Figure S1.

To examine whether the four clusters identified above from the expanded GBM dataset, corresponded to clusters observed in previous studies [7], we constructed a confusion table comparing genes up regulated in each subtype with the 840 genes (210 genes for each cluster) previously identified in [7]. To do so, for each subtype we identified genes that were up regulated with a fold change of at least two compared to the mean expression level of samples in all other subtypes. Because the cluster signatures introduced in the previous studies represented mostly the up regulated genes in each subtype, we only examined those genes that were over-expressed. We observed a close correspondence between the clusters we characterized and those introduced previously (Table 2). In the remainder of the paper, we use the same names for the studied subtypes.

**Table 2:** Confusion table for the genetic signatures identified in this study and the previous study (Verhaal *et al*. 2010).

	Proneural	Neural	Classical	Mesenchymal
**Proneural (Verhaal ** ***et al*** **.)**	59	9	1	0
**Neural (Verhaal ** ***et al*** **.)**	6	21	0	0
**Classical (Verhaal ** ***et al*** **.)**	0	0	23	0
**Mesenchymal (Verhaal ** ***et al*** **.)**	0	0	0	69

#### Expression of EMT signature among GBM subtypes

**Figure 3:**
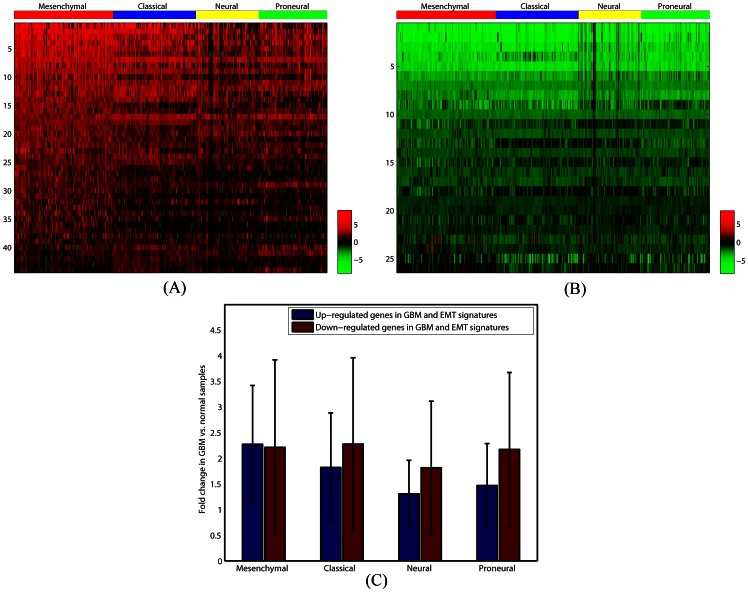
Logarithm base two of GBM vs. Normal fold changes of genes that are similarly expressed in the genetic signature of EMT and GBM subtypes. (A) Heatmap of the GBM vs. Normal fold changes of genes that are up regulated in the genetic signature of EMT and also in the genetic signature of at least one of the GBM subtypes. Fold changes are given separately for each GBM sample. (B) Heatmap of the GBM vs. Normal fold changes of genes that are down regulated in the genetic signature of EMT and in the genetic signature of at least one of the GBM subtypes. As before, fold changes are given separately for each GBM sample. (C) Logarithm base two GBM vs. Normal fold changes in Figures (A) and (B) are averaged over all genes and samples in each subtype of GBM. The result is presented for both up regulated and down regulated genes. In the case of down regulated genes, absolute values of the average log base two fold changes are shown. As presented, the average GBM vs. Normal fold change of up regulated genes decreased as we cross from the mesenchymal subtype to the other three subtypes. However, the average GBM vs. Normal fold changes of down regulated genes were almost identical in all GBM subtypes.

We then attempted to determine whether the expression of EMT related genes varies among these GBM molecular subtypes. For each subtype, we identified genes with at least a two-fold change in expression compared with normal samples (File S2). We observed that genes that were up regulated in EMT were significantly correlated with those that were up regulated among *all* GBM subtypes. Table 3 presents the number of genes in the up regulated signature as well as length of the overlap between them. It also provides length of the overlaps that we expect due to pure chance and the TSFET p-values evaluating the significance of the observed overlaps. Although up regulated genes in the EMT signature had significant overlaps with the signature of all GBM subtypes, we noted that the number of up regulated genes and their expression levels decreased as we crossed from the mesenchymal subtype to the other three subtypes (Figures 3A and 3C). We also observed that genes that were down regulated in EMT were significantly correlated with those that were down regulated among all GBM subtypes (Table 3). Interestingly, in contrast to the up regulated genes, the expression levels of down regulated genes were almost identical in all four of the GBM subtypes (Figures 3B and 3C). Finally, we compared signatures with opposite expressions in the signatures of EMT and GBM subtypes. The results of these comparisons are provided in Table 3. As demonstrated in this table, the overlaps between signatures of EMT and GBM subtypes with the opposite direction are either not statistically significant or less than what is expected due to chance. These results, taken together, support the existence of a direct relationship between signatures of EMT and GBM subtypes. Table S3A lists genes that are up regulated in EMT and at least one of the GBM subtypes, as well as their GBM-Normal folds of change in each of the four GBM subtypes. Similarly, Table S3B presents genes that are down regulated in EMT and at least one of the GBM subtypes, together with their GBM-Normal folds of change in each of the GBM subtypes. The sets of genes with reverse expression in EMT and GBM sample are provided in Tables S3C and S3D.

**Table 3:** Comparison of EMT and GBM subtypes.

Size of background set: 11296	Overlap (Exp. Overlap)	p-value
**EMT^up^(78)- Mes^up^(1610)**	40 (11.12)	9.65×10^−15^
**EMT^up^(78)- Classical^up^(1456)**	34 (10.05)	1.83×10^−11^
**EMT^up^(78)- Neural^up^(1174)**	29 (8.11)	2.99×10^−10^
**EMT^up^(78)- Proneural^up^(1417)**	29 (9.78)	2.38×10^−8^
**Size of background set: 11296**	**Overlap (Exp. Overlap)**	**p-value**
**EMT^down^(129)-Mes^down^(1098)**	21 (12.54)	1.62×10^−2^
**EMT^down^(129)-Classical^down^(1040)**	24 (11.88)	9.98×10^−4^
**EMT^down^(129)-Neural^down^(837)**	17 (9.56)	1.73×10^−2^
**EMT^down^(129)-Proneural^down^(951)**	23 (10.86)	5.79×10^−4^
**Size of background set: 11296**	**Overlap (Exp. Overlap)**	**p-value**
**EMT^down^(129)-Mes^up^(1610)**	14 (18.39)	3.11×10^−1^
**EMT^down^(129)-Classical^up^(1456)**	7 (16.63)	7.80×10^−3^
**EMT^down^(129)-Neural^up^(1174)**	7 (13.41)	7.93×10^−2^
**EMT^down^(129)-Proneural^up^(1417)**	2 (16.18)	1.11×10^−5^
**Size of background set: 11296**	**Overlap (Exp. Overlap)**	**p-value**
**EMT^up^(78)-Mes^down^(1098)**	7 (7.58)	1.00
**EMT^up^(78)-Classical^down^(1040)**	8 (7.18)	6.94×10^−1^
**EMT^up^(78)-Neural^down^(837)**	7 (5.78)	5.17×10^−1^
**EMT^up^(78)-Proneural^down^(951)**	10 (6.57)	1.53×10^−1^

Two-sided Fisher exact test (TSFET) is used to assess significance of the overlap between the genetic signature of EMT and each of the GBM subtypes. The background set in TSFET includes genes whose expression levels are provided in the data sets taken from both TCGA and (12). The number of these genes in the background set was 11296. The number of genes in each signature is provided in the parentheses in the first column. The second column provides length of the overlap between the signatures as well as length of the overlap that we expect due to pure chance. The third column provides the TSFET p-values evaluating the significance of the overlap between the signatures.

**Figure 4:**
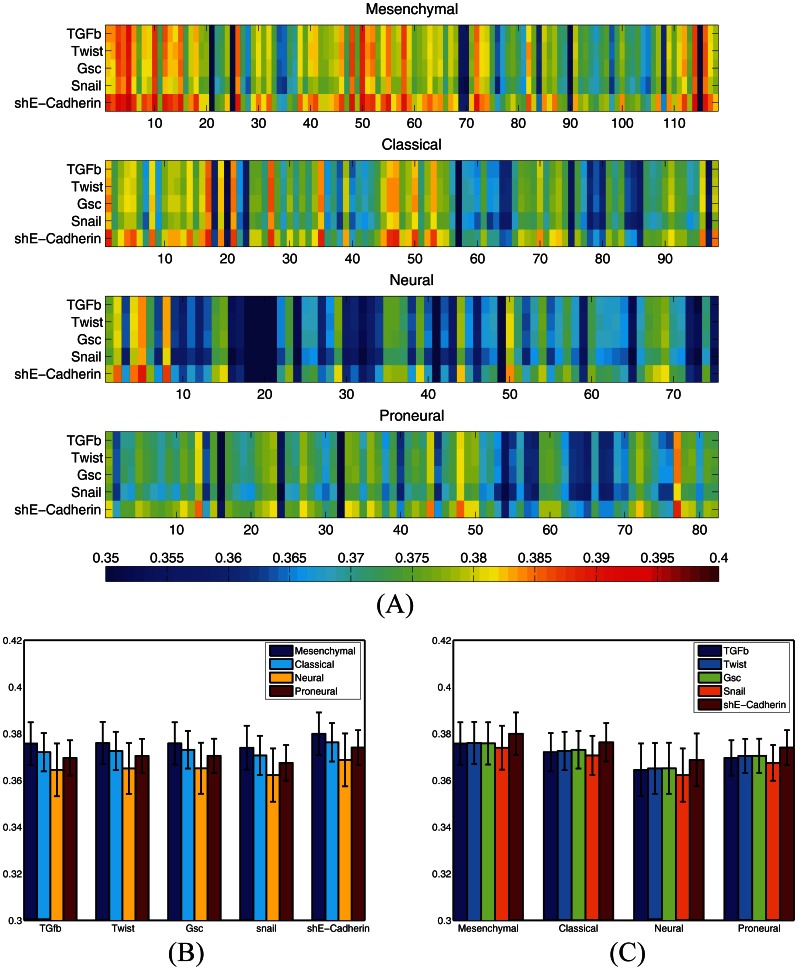
Pearson correlation between genetic signature of GBM samples and genetic signature of cells which are forced to undergo an EMT using 5 different inducers, namely, TGFβ, Twist, Gsc, Snail and shE-Cadherin. (A) Pearson correlation between individual GBM samples from four GBM subtype and cells which are forced to undergo an EMT. (B) Pearson correlation between GBM subtypes and EMT signatures averaged over samples in each GBM subtype. As demonstrated, the mesenchymal subtype has the highest correlation with the EMT signatures compared to the other three subtypes. (C) Pearson correlation between GBM subtypes and EMT signatures averaged over samples obtained from each of the five EMT inducers. As demonstrated, the genetic signature of samples exposed to shE-Cadherin has the highest correlation with the signature of GBM samples.

The core EMT signature was determined from the intersection of gene signatures obtained after exposure of cells to five different EMT inducers [12]. We sought to determine whether the specific EMT inducer used to transform cells influenced the similarity between GBM and EMT gene expression. We evaluated the Pearson correlation between gene expression signature for each of the TCGA GBM samples and each of the five representative gene expression patterns corresponding to five EMT inducers (Figure 4A–C). As demonstrated, the mesenchymal subtype of GBM has the highest correlation with all EMT inducers (Figure 4B). In addition, the correlation between the signatures obtained from shE-Cadherin and the GBM samples is larger than the correlation between other EMT inducers and GBM samples. This order holds for all GBM subtypes (Figure 4C).

### Negative Correlation of CD133 Genes with the Core EMT Signature

We sought to determine whether there was a significant relationship between the expression of CD133 and the EMT process. We sorted two primary human GBM samples (BT1 and BT2) and two normal fetal brain samples (N1 and N2) for CD133 expression (Overall eight samples, four positive and four negative; see Methods and Ref. [22]). We identified those genes that were either up or down regulated with at least two-fold change in each CD133+ sample when compared to the corresponding CD133− sample. We observed statistically significant *negative* correlation between the expression of EMT and CD133 genes. That is, genes that were down regulated during the EMT process have a strong correlation with genes that were up regulated in all of the CD133+ samples (Table 4). In addition, genes that were up regulated in EMT exhibited a significant correlation with genes that were down regulated in the BT2 and N2 CD133+ samples (Table 4). We noted that the size of overlap between signatures of CD133 and EMT with the same direction is either not statistically significant or less than what is expected due to chance (Table 4). These results taken together demonstrate a negative correlation between signatures of EMT and CD133. Table 4 presents the number of genes in the EMT and CD133 signatures as well as length of the overlap between them. It also provides length of the overlap that we expect due to pure chance. The background set in the TSFET in this case includes genes whose expression values were provided in both the dataset taken from [12] and our own data. Table S4A presents genes that are down regulated in EMT and up regulated in at least 2 out of 4 CD133+ samples. Table S4B lists genes that are up regulated in EMT and down regulated in at least 2 out of 4 CD133+ samples. File S3 presents the CD133 signatures obtained from each of the four CD133+/− pairs.

**Table 4:** Comparison of EMT and CD133 signatures.

Size of background set: 11774	Overlap (Exp. Overlap)	p-value
EMT^down^(132)-BT1+^ up^(486)	14 (5.4486)	1.1×10^−3^
EMT^down^ (132)-BT2+^ up^(377)	15 (4.2266)	2.05×10^−5^
EMT^down^ (132)-N1+^ up^(480)	25 (5.3813)	9.93×10^−11^
EMT^down^ (132)-N2+^ up^(836)	64 (9.3725)	1.110×10^−38^
**Size of background set: 11774**	**Overlap (Exp. Overlap)**	**p-value**
EMT^up^(77)-BT1+^ down^(191)	1 (1.2491)	1.00
EMT^up^(77)-BT2+^ down^(50)	1 (0.3270)	2.80×10^−1^
EMT^up^(77)-N1+^ down^(77)	1 (0.5036)	3.98×10^−1^
EMT^up^(77)-N2+^ down^(267)	14 (1.7461)	1.59×10^−9^
**Size of background set: 11774**	**Overlap (Exp. Overlap)**	**p-value**
EMT^up^(77)-BT1+^ up^(107)	1 (3.1784)	3.790×10^−1^
EMT^up^(77)-BT2+^ up^(1197)	3 (2.4655)	7.381×10^−1^
EMT^up^(77)-N1+^ up^(857)	1 (3.1391)	3.773×10^−1^
EMT^up^(77)-N2+^ up^(811)	0 (5.4673)	6.2×10^−3^
**Size of background set: 11774**	**Overlap (Exp. Overlap)**	**p-value**
EMT^down^(132)-BT1+^ down^(860)	1 (2.1413)	7.27×10^−1^
EMT^down^(132)-BT2+^ down^(37)	1 (0.5606)	4.32×10^−1^
MT^down^(132)-N1+^ down^(177)	2 (0.8633)	2.14×10^−1^
EMT^down^(132)-N2+^ down^(1108)	2 (2.9934)	7.72×10^−1^

Two-sided Fisher exact test (TSFET) is used to assess significance of the overlap between EMT and CD133 signatures. The background set in TSFET includes genes whose expression levels are provided in the data sets taken from both (12) and the present study. The number of genes in the background set is equal to 11774. The number of genes in the EMT and CD133 signatures is provided within the parentheses in the first column. The second column provides length of the overlap between the signatures as well as length of the overlap that we expect due to pure chance. The third column provides the TSFET p-values evaluating the significance of the overlap between the signatures. In this table, the overlap between the signatures is not statistically significant if p-value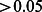.

### Correlation of CD133 Gene Signatures and GBM Subtypes

We sought to compare genes that were up or down regulated in the CD133 signatures with the genes that were up or down regulated in the TCGA GBM subtypes. We used the CD133 signatures obtained in the previous section, i.e., two signatures from GBM CD133+/− pairs, and two signatures from normal CD133+/− pairs. For each TCGA GBM subtype, we examined those genes having at least a two-fold difference in expression when compared to the normal samples (File S2). We aimed to see how the genes representative of each GBM subtype correlate with the CD133 signatures. We noted that there are some differences between CD133 signatures obtained from GBM and normal samples in terms of how they are related to the signatures of GBM subtypes. Because of this, we present the results corresponding to CD133 signatures obtained from GBM and normal samples separately.

We call the set of two CD133 signatures obtained from GBM samples CD133_BT_ signatures. Similarly, we call the set of CD133 signatures obtained from the two normal samples N1 and N2, CD133_N_ signatures. The results of comparison between signatures of GBM subtypes and CD133_BT_ signatures are provided in Table 5. It presents length of the intersection between genes that are up regulated in the signatures of GBM subtypes and CD133_BT_. It also provides length of the intersections that we expect due to chance and the p-values assessing significance of the intersections. Table 5 also provides similar results for genes that are down regulated in the signatures of GBM subtypes and CD133_BT_. The results obtained from comparing signatures of GBM and CD133_N_ with opposite directions are provided in Table 5. All p-values in Table 5 are obtained based on two-sided Fisher exact test with a background set consisting of all genes whose expression levels are provided in both TCGA and our data.

**Table 5:** Comparison between genetic signatures of CD133 obtained from brain tumor samples BT1 and BT2 and genetic signatures of GBM subtypes.

Size of background set: 11632	BT1^un^(486)	BT2^up^(382)
Mes^up^(1659)	1.38×10^−55^	2.72×10^−53^
	208 (69.32)	177 (54.48)
Classical^up^(1511)	1.17×10^−20^	1.04×10^−2^
	139 (63.13)	67 (49.62)
Neural^up^(1212)	3.54×10^−35^	5.97×10^−28^
	147 (50.64)	116 (39.80)
Proneural^up^(1463)	3.37×10^−16^	1.58×10^−1^
	126 (61.13)	57 (48.05)
**Size of background set: 11632**	**BT1^down^(184)**	**BT2^down^(47)**
Mes^down^(1128)	8.28×10^−24^	8.03×10^−1^
	68 (17.84)	5 (4.56)
Classical^down^(1059)	4.53×10^−23^	6.14×10^−1^
	65 (16.75)	5 (4.28)
Neural^down^(850)	4.37×10^−21^	5.80×10^−1^
	56 (13.45)	2 (3.43)
Proneural^down^(963)	3.02×10^−19^	7.94×10^−1^
	57 (15.23)	4 (3.89)
**Size of background set: 11632**	**BT1^up^(486)**	**BT2^up^(382)**
Mes^down^(1128)	1.44×10^−06^	1.46×10^−4^
	19 (47.13)	17 (37.04)
Classical^down^(1059)	2.15×10^−3^	5.87×10^−1^
	26 (44.25)	31 (34.78)
Neural^down^(850)	2.97×10^−6^	1.59×10^−2^
	12 (35.51)	16 (27.91)
Proneural^down^(963)	5.32×10^−3^	5.71×10^−1^
	24 (40.24)	28 (31.63)
**Size of background set: 11632**	**BT1^down^(184)**	**BT2^down^(47)**
Mes^up^(1659)	1.35×10^−3^	2.05×10^−1^
	12 (26.24)	10 (6.70)
Classical^up^(1511)	2.68×10^−3^	8.28×10^−1^
	11 (23.90)	5 (6.11)
Neural^up^(1212)	8.81×10^−2^	1.00
	12 (19.17)	5 (4.90)
Proneural^up^(1463)	4.78×10^−3^	5.12×10^−1^
	11 (23.14)	4 (5.91)

Two-sided Fisher exact test (TSFET) is used to assess significance of the overlap between genetic signatures of CD133 and GBM subtypes. The background set in TSFET includes genes whose expression levels are provided in the data sets taken from TCGA and the present study. The number of genes in the background set is equal to 11632. The number of genes in the signatures of GBM subtypes and CD133 is provided within the parentheses in the first column and first row, respectively. There are two rows in each of the cells in the body of the table. The first row denotes length of the overlap between the genetic signatures as well as length of the overlap that we expect due to pure chance. The second row denotes the p-value assessing the significance of the overlap between the signatures. The overlap between the signatures is not statistically significant if p-value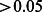.

As illustrated in Table 5, length of overlap between signatures of GBM subtypes and CD133_BT_ with the same direction is larger than what is expected due to chance whenever the results are significant. Also, it can be seen that the length of overlap between signatures of GBM subtypes and CD133_BT_ with the opposite direction is less than what is expected due to chance whenever the results are significant (Table 5). These results, taken together, indicate that there is a direct relationship between signatures of GBM subtypes and CD133_BT_.

Table 6 presents the results of comparison between signatures of GBM subtypes and CD133_N_. It compares signatures of GBM subtypes and CD133_N_ with the same direction. As demonstrated, except for the case of intersection between genes up regulated in both N2 and Proneural subtype of GBM, lengths of the overlap between signatures of GBM subtypes and CD133_N_ with the same direction are larger than what is expected due to chance. In this sense, CD133_N_ signatures are similar to CD133_BT_ signatures. Table 6 compare signatures of GBM subtypes and CD133_N_ with the opposite direction. As demonstrated in this table, in spite of what we observed for CD133_BT_ signatures, the length of overlap between signatures of GBM subtypes and CD133_N_ with the opposite direction is either not statistically significant or larger than what is expected due to chance.

**Table 6:** Comparison between genetic signatures of CD133 obtained from normal samples N1 and N2 and genetic signatures of GBM subtypes.

Size of background set: 11632	N1^up^(475)	N2^up^(834)
Mes^up^(1698)	2.94×10^−29^	4.79×10^−11^
	163 (67.75)	187 (118.95)
Classical^up^(1548)	2.12×10^−2^	8.31×10^−1^
	79 (61.70)	106 (108.34)
Neural^up^(1239)	1.09×10^−20^	7.86×10^−12^
	119 (49.49)	150 (86.90)
Proneural^up^(1502)	8.88×10^−1^	3.95×10^−2^
	58 (59.74)	86 (104.90)
**Size of background set: 11632**	**N1^down^(70)**	**N2^down^(256)**
Mes^down^(1128)	2.19×10^−1^	8.70×10^−2^
	10 (6.79)	33 (24.83)
Classical^down^(1059)	1.41×10^−1^	1.50×10^−2^
	10 (6.37)	35 (23.31)
Neural^down^(850)	1.69×10^−1^	6.03×10^−4^
	8 (5.12)	34 (18.71)
Proneural^down^(963)	7.85×10^−2^	5.61×10^−3^
	10 (5.80)	34 (21.19)
**Size of background set: 11632**	**N1^up^(475)**	**N2^up^(834)**
Mes^down^(1128)	9.37×10^−1^	8.95×10^−12^
	45 (46.06)	142 (80.88)
Classical^down^(1059)	2.21×10^−1^	1.30×10^−11^
	51 (43.24)	135 (75.93)
Neural^down^(850)	5.29×10^−1^	1.04×10^−8^
	38 (34.71)	106 (60.94)
Proneural^down^(963)	4.44×10^−1^	4.28×10^−11^
	44 (39.32)	124 (69.05)
**Size of background set: 11632**	**N1^down^(70)**	**N2^down^(256)**
Mes^up^(1698)	8.64×10^−1^	7.18×10^−1^
	9 (9.98)	34 (36.51)
Classical^up^(1548)	3.71×10^−1^	3.49×10^−1^
	6 (9.09)	28 (33.25)
Neural^up^(1239)	6.97×10^−1^	7.82×10^−2^
	8 (7.29)	18 (26.67)
Proneural^up^(1502)	3.70×10^−1^	5.60×10^−2^
	6 (8.80)	22 (32.20)

Two-sided Fisher exact test (TSFET) is used to assess significance of the overlap between genetic signature of CD133 and GBM subtypes. The background set in TSFET includes genes whose expression levels are provided in the data sets taken from TCGA and the present study. The number of genes in the background set is equal to 11632. The number of genes in the signatures of GBM subtypes and CD133 is provided within the parentheses in the first column and first row, respectively. There are two rows in each of the cells in the body of the table. The first row denotes length of the overlap between the genetic signatures as well as length of the overlap that we expect due to pure chance. The second row denotes the p-value assessing the significance of the overlap between the signatures. In the table, the overlap between the signatures is not statistically significant if p-value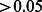.

In the next step, we sought to see whether there are any differences among the four subtypes of GBM in terms of their relation with the CD133 signatures. From Tables 5 and 6 it can be seen that, in terms of genes that are up regulated in the signatures of both GBM and CD133, signatures of *mesenchymal* and *neural* subtypes are more strongly correlated with the signatures of CD133 compared to *proneural* and *classical* subtypes. In particular, it can be seen that the length of overlap between signatures of *mesenchymal* and *neural* subtypes and CD133 signatures is always larger than what is expected due to chance. From the same tables it can be seen that, in the statistically significant cases, the overlap between genes that are up regulated in the signatures of CD133 and signatures of *proneural* and *classical* subtypes are either larger than what is expected due to chance with a weaker p-value compared to mesenchymal and neural subtypes or less than what is expected due to chance. This difference between mesenchymal and neural subtypes and the proneural and classical subtypes is consistently observed in all four signatures of CD133. One can see that such a clear difference is not observed between GBM subtypes in terms of genes that are down regulated in the signatures of GBM subtypes and CD133, or genes that are expressed with reverse direction in the signatures of GBM subtypes and CD133.

**Figure 5:**
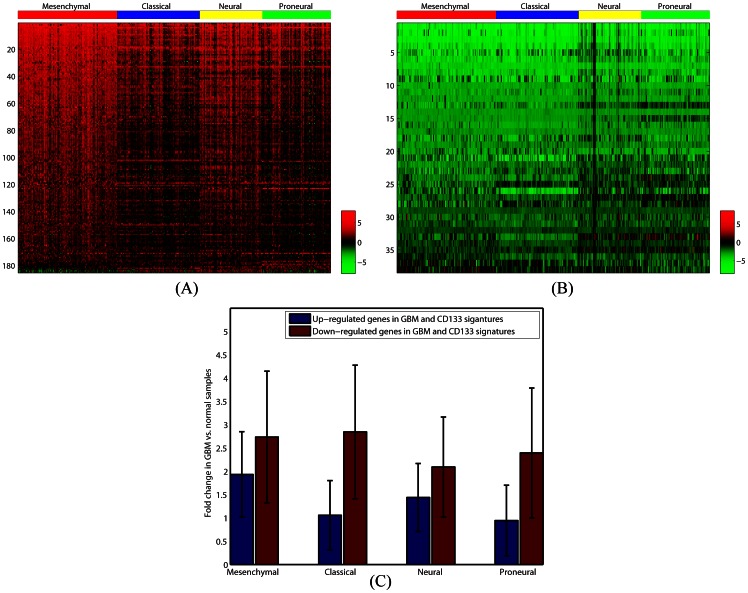
Logarithm base two of GBM vs. Normal fold change of genes that are similarly expressed in the genetic signature of CD133 and GBM subtypes. (A) Heatmap of the GBM vs. Normal fold changes of genes that are up regulated in the genetic signature of CD133 and in at least one of the GBM subtypes with a fold change of at least two. Fold changes are given separately for each GBM sample. (B) Heat map of the GBM vs. Normal fold changes of genes that are down regulated in the genetic signature of CD133 and in at least one of the GBM subtypes with a fold change of at least two. (C) Logarithm base two of GBM vs. Normal fold changes are averaged over all genes and samples in each subtype of GBM. The result is presented for both up regulated and down regulated genes. In the case of down regulated genes, absolute values of the average log base two fold changes are shown. As presented, the average GBM vs. Normal fold change of genes up regulated in the CD133 signature is higher in the mesenchymal and neural subtypes when compared to the classical and proneural subtypes. However, average GBM vs. Normal fold change of down regulated genes in the CD133 signature does not vary significantly among GBM subtypes.

To further explore the relationship between signatures of GBM subtypes and CD133, we identified a signature for CD133 using genes that are either up or down regulated in at least two of the four CD133 signatures. Figures 5A and 5C illustrates GBM vs. normal fold-change of genes that are up regulated in the CD133 signature and in at least one of the GBM subtypes. Similarly, Figure 5B and 5C illustrate GBM vs. normal fold-changes of genes that are down regulated in the CD133 signature and in at least one of the GBM subtypes. As illustrated in Figures 5A and 5C, the mesenchymal and neural subtypes of GBM demonstrate a higher correlation with the CD133 signature in terms of the up regulated genes. Consistent with the results presented in Tables 5 and 6, GBM subtypes do not demonstrate significant differences in terms of genes that are down regulated in the CD133 and GBM signatures (Figures 5B and 5C). Table S5 lists genes with both similar and reverse expressions in the signature of GBM subtypes and CD133 cell surface protein.

#### Relation between mesenchymal subtype of GBM, EMT signature and CD133 signature

In this study, in addition to genes with similar expression in the signature of the mesenchymal subtype and each of the EMT and CD133 signatures, we recorded genes with reverse expression in the genetic signature of the mesenchymal subtype and each of the above signatures (Tables S3 and S5). Moreover, when comparing EMT and CD133 signatures, we observed that the genetic signatures of EMT and CD133 are negatively correlated with each other. We sought to see whether the differences between the mesenchymal subtype of GBM and EMT signatures can be at least partly explained by the similarity between the mesenchymal signature and the CD133 signature. In other words, we aimed to determine whether part of the differences between the mesenchymal subtype of GBM and EMT signature can be attributed to the fact that the mesenchymal subtype of GBM has correlations with the CD133 signature whereas EMT and CD133 signatures are two opposing signatures.

Similarly, we sought to see whether the differences between the mesenchymal subtype of GBM and CD133 signatures can be partly explained in light of the similarity between the mesenchymal subtype and EMT signatures.

As for the first part, the differences between the mesenchymal subtype of GBM and the EMT signature include genes that are up regulated in the mesenchymal subtype and down regulated in the EMT signature, and genes that are down regulated in the mesenchymal subtype and up regulated in the EMT signature. We denote these gene lists by EMT^down^-Mes^up^ and EMT^up^-Mes^down^, respectively. The above hypothesis indicates that genes in the EMT^down^Mes^up^ set are down regulated in the EMT signature but up regulated in the mesenchymal subtype perhaps because they are up regulated in the CD133 signature. To validate this hypothesis, we sought to see whether the genes in the EMT^down^-Mes^up^ set significantly coincide with genes that are up regulated in CD133 signature. Similarly, we sought to see whether the genes in the EMT^up^-Mes^down^ set has a significant overlap with genes down regulated in CD133 signature, explaining why they are down regulated in the mesenchymal subtype instead of being up regulated. Moreover, to address the differences between the mesenchymal subtype and CD133 signatures, we assessed the significance of the correlation between CD133^down^Mes^up^ and EMT^up^ genes, and also between CD133^up^-Mes^down^ and EMT^down^ genes. In this section, to obtain signatures of EMT, GBM subtypes and CD133 we used a threshold of 0.5 instead of 1 in the logarithm base two scale to identify differentially expressed genes in each group. That is because in this section we needed to obtain intersection of three genetic signatures all together, and we wanted the length of these gene lists to be large enough so that their intersection is non-empty. Table 7 lists length of the intersections between the above gene lists as well as length of the overlap that we expect due to chance. In addition, it presents p-values obtain from TSFETs, and list of the genes at the intersection of the above gene lists. The background set in the TSFET in this case includes genes whose expression values are given in the datasets taken from studies [12], [7] and our own study. The total number of genes in the background set was 10986. As demonstrated in Table 7 all p-values are small, indicating that at least part of the differences between the genetic signature of the mesenchymal subtype of GBM and EMT and CD133 signatures may be due to the fact that the genetic signatures of EMT and CD133 are inversely correlated with each other.

**Table 7:** Justification of the difference between the genetic signature of mesenchymal subtype of GBM and the EMT and CD133 signatures.

	Overlap (Expected)	p-values
**EMT^down^-Mes^up^ (13) and CD133^up^(620)**	24 (5.77)	1.24×10^−10^
**EMT^up^-Mes^down^(7) and CD133^down^(174)**	3 (0.87)	5.36×10^−2^
**CD133^down^-Mes^up^(16) and EMT^up^(76)**	14 (1.46)	1.35×10^−10^
**CD133^up^-Mes^down^(61) and EMT^down^(127)**	14 (4.27)	9.32×10^−5^

It was hypothesized that the set of genes that are down-regulated in the EMT signature but are up-regulated in the mesenchymal subtype significantly coincide with genes that are up-regulated in the CD133 signature. Two-sided Fisher exact test (TSFET) was used to validate the significant of overlap between the above set of genes (denoted by EMT^down^-Mes^up^) and the genes up-regulated in the CD133 signature (denoted by CD133^up^). Following the same idea, two-sided Fisher exact test was used to evaluate the significant of overlap between the EMT^up^-Mes^down^ and CD133^down^ sets, the CD133^down^-Mes^up^ and EMT^up^ sets, and the CD133^up^-Mes^down^ and EMT^down^ sets. The length of the gene lists as well as overlaps between them is provided in the table below. Also, length of the overlaps that is expected due to chance and p-values assessing significance of the overlaps are presented. Moreover, genes at the intersection between the gene lists are given. The background set of genes in the TSEFT includes gene whose expression levels are provided in the data sets taken from TCGA, (12) and the present study. The number of genes in the background set is equal to 10986. As before, the overlap between the signatures is not statistically significant if p-value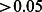.

## Discussion

GBM is the most common and lethal brain tumor in humans with median survival of 6–12 months. Studies on GBM have undergone two major developments in recent years. The first includes reports on the mesenchymal transition in glioblastoma tumors and the correlation of this transition with the tumor severity [5]. Moreover, classification of GBM samples demonstrated the existence of a group whose genetic signature is related to mesenchymal cells. The second development is the stem cell hypothesis indicating that a small subset of cells in the tumor are sufficient for tumor initiation and contribute to resistance [16], [19]. The cell surface protein CD133 is proposed as a putative marker for identification of this subset.

To address the complexities of GBM, genome studies have been widely applied to study this disease at a molecular level. In this paper, we use genetic studies to bridge the gap between the genetic attributes of GBM cells and each of the above developments. The three sources of data used in our study include: TCGA GBM samples, EMT-induced epithelial cells in Taube *et al.* [12], and GBM and normal data sorted by CD133 cell surface marker (see Methods).

We first identified an overall signature for GBM by comparing the GBM and normal data of TCGA. The top genes identified by comparing TCGA GBM and normal samples reflected the status of various properties of GBM samples as a result of inflammation, coagulation, extracellular matrix remodeling, and angiogenesis, as well as several genes associated with a mesenchymal phenotype consistent with previous reports [5], [6]. We also used the recently identified core EMT signature for identifying important genes in the EMT process. We observed a significant overlap between those genes that were either up or down regulated in both the EMT and GBM samples. Some genes from the list have been already reported: for instance POSTN and collagen-specific genes [6]. We used the NCI Cancer Molecular Analysis (CMA) Portal to check the top genes in the EMT signature for their contribution to the survival of patients with GBM. The following genes in the EMT signature were predictors of patients’ survival (ordered from lower, 0.0003, to higher, 0.2, p-values): TAGLN2, IGFBP2, POSTN, TNC, SERPINA3, IGFBP3, TGFBI, COL4A1, and HMOX1. Interestingly, IGFBP has been recently reported as one of the 9-gene signature specifying poor-prognosis GBMs [25].

GBM is known to be a heterogeneous cancer. Recently, four subtypes of this cancer have been identified: mesenchymal, classical, neural and proneural. We sought to compare the correlation between the EMT signature and each of the above GBM subtypes. We used the two-sided Fisher exact test to assess the overlap between genes that are up/down regulated in EMT signature and the GBM subtypes. We observed a significant overlap between the genes that are up regulated in the EMT signature and those that are up regulated in each of the GBM subtypes. In addition, a significant correlation was observed between genes that are down regulated in the EMT signature and also down regulated in each of the GBM subtypes. We observed that the number of up regulated genes and their expression levels decrease as we crossed from the mesenchymal subtype to the other three subtypes. However, the number of down regulated genes and their expression levels remain almost constant for all four subtypes. These findings indicate that although all GBM subtypes have some degree of similarity with the EMT process, the mesenchymal subtype has the closest ties. The core EMT signature used in this study was obtained from the intersection of gene signatures of five different EMT inducers: TGF­β, Twist, Gsc, Snail and shE-Cadherin. We studied the correlation of the GBM subtypes with each of the above EMT-inducers, exclusively. The studied EMT inducers demonstrated slightly different levels of similarity with the GBM samples. Among them shECadherin-induced samples had the highest correlation with the GBM samples. On the other hand, the mesenchymal subtype of GBM consistently demonstrated the highest correlation with the samples obtained from all EMT inducers.

Motivated by recent results indicating that the mesenchymal subtype includes mostly CD133− samples, we compared the core signature of EMT with the four signatures we derived for CD133. We observed a significant overlap between the genes up (down) regulated in EMT signature and those down (up) regulated in CD133 signatures. This is indicative of a negative correlation between signatures of EMT and CD133.

Finally, we compared the genetic signature of CD133 with each of the GBM subtypes. We noticed that the CD133 signature demonstrates a significant positive correlation with mesenchymal and neural subtypes in both GBM- and normal-sample-derived CD133 signatures, and that the CD133 signature is less correlated with the classical and proneural subtyupes. This is consistent with the results of Verhaak *et al*. [7], indicating that the mesenchymal and neural subtypes have correlations with *astroglia* and *neuronal* cells, and the classical and proneural subtypes have correlations with *astrocytic* and *oligodentrocytic* cells. It is known that glia and neuronal cells are immediate descendants of neural stem cells, while astrocytic and oligodentrocytic cells are more mature in the hierarchy.

A recent paper by Yan *et al.* [19] indicates that the proneural subtype is the only subtype which significantly correlates with CD133 signature, and its correlation is positive. Our findings support a positive correlation between genetic signature of proneural subtype and signatures of CD133 obtained from GBM samples. However, as opposed to the findings presented there, our data suggest that mesenchymal subtype of GBM more significantly correlates with the CD133 signature. Given the evidence on stemness properties of CD133+ samples, similarity to CD133 signature may be a sign for the stem cell origin and poor prognosis of this subtype.

Although we observed that the mesenchymal subtype has strong correlations with both EMT and CD133 signatures, we also observed a subset of genes with opposite expressions in mesenchymal subtype and each of the above signatures. We noticed that at least part of this difference can be attributed to the fact that signatures of EMT and CD133 are negatively correlated with each other, and thus similarity of the mesenchymal subtype with both of them will be at the expense of developing some differences with the signature of the other phenotype.

In this study, we noted that the CD133 signatures obtained from normal samples are different from CD133 signatures obtained from GBM sample in terms of their relation with signatures of GBM samples with reverse direction. In particular, we noted that the overlap between signatures of GBM subtypes and CD133_BT_ is less than what is expected due to chance whenever the results are significant, while the overlap between signatures of GBM subtypes and CD133_N_ is larger than what is expected due to chance whenever the results are significant. Once explanation for this behavior is that, as demonstrated in Table 4, signatures of CD133 obtained from normal samples have a stronger negative correlation with the signatures of EMT. As a result, they demonstrate stronger differences with the signatures of GBM subtypes which are directly related to the signature of EMT.

We observed that the mean expression of the suppressor protein PROM1 (CD133) in the mesenchymal GBM subtype was lower than its mean expression level in the other three subtypes (mean expression levels in mesenchymal, classical, neural and proneural subtypes are 6.46, 7.13, 7.37 and 8.09, respectively). This observation supports the results obtained by Chen *et al*. [26], showing that the mesenchymal subtype of GBMs mostly included CD133− cells. Recent studies suggest that the expression of CD133 is controlled by epigenetic factors [27]. It is interesting to study the expression of CD133 and its product on the cell surface, however, our results suggest that one should differentiate between the similarity between the signature of a gene, or the gene itself, with a given process. We observed that while the signature of CD133 is highly correlated with the signature of the mesenchymal subtype, the PROM1 gene itself is less expressed in the mesenchymal subtype when compared to other subtypes of GBM.

Deciphering the molecular mechanisms underlying the origin and progression of each subtype of glioblastoma will enable more accurate prognoses to be made and help in the development of more potent therapies. The results presented herein further this goal by tracking the links (similarities and differences) between glioblastoma subtypes and each of the EMT and CD133 signatures.

## Methods

### Ethics Statement

Human brain tumor samples were obtained from patients through written informed consent, as approved by the Hamilton Health Sciences/McMaster Health Sciences Research Ethics Board. All animal work was conducted under an Animal Utilization Protocol (08-03-06) reviewed and approved by the McMaster University Animal REB and by the Hamilton Health Sciences/McMaster Health Sciences Research Ethics Board.

### Primary Cell and Sphere Culture

Human brain tumor and human fetal brain samples were obtained from consenting patients, as approved by the Hamilton Health Sciences/McMaster Health Sciences Research Ethics Board. Samples were dissociated in artificial cerebrospinal fluid containing 0.2 Wunisch Unit/ml Liberase Blendzyme® 3 (Roche), and incubated at 37°C in a shaker for 30 minutes. The dissociated tissue was then filtered through 70 µm cell strainer and collected by centrifugation at 1500 rpm for 3 min. Tumor cells were resuspended in Tumor Sphere Medium (TSM) consisting of a chemically defined serum-free neural stem cell medium [28], [29], [30], human recombinant EGF (20 ng/ml: Sigma), bFGF (20 ng/ml; Invitrogen), LIF (10 ng/ml; Chemicon), Neural Survival Factor (NSF) (1×; Clonetics), N-acetylcysteine (60 ug/ml: Sigma) and antibiotic antimycotic solution. Red blood cells were removed using RBC lysis buffer (Stem Cell Technology). All experiments were performed following minimal culture.

### Magnetic Cell Sorting and Flow Cytometry (FACS) Analysis

Primary spheres were dissociated with Liberase Blendzyme 3 to single cell suspension. CD133+ cells were purified by magnetic activated cell sorting columns (MACS; Miltenyi Biotec) using microbeads conjugated to CD133 antibodies. The percentage expression of each group of unsorted cells, CD133+ selected cells and the negative fraction was determined by FACSCalibur (BD bioscicences) using APC-labeled anti-CD133 antibodies (Miltenyi). The purity of CD133+ cell fractions from brain tumors was 85±3.4%, and the CD133− cell fraction purity was 99.5±0.3%. For human NSC, the purity of CD133+ cells was 94±4.6%, and CD133− cell fraction purity was 99.3±0.4%. The appropriate isotype control served as the negative control for every experiment.

### Microarray

Total RNA was extracted from sorted CD133+ and CD133− populations from BT and human NSC samples using Qiagen RNeasy Micro kit (Qiagen). Total RNA was submitted to the University Health Network Microarray Centre (Toronto, ON). Prior to target labeling, the purity of every sample was evaluated using a 20 ng aliquot with a 2100 Bioanalyzer (Agilent Technologies). Purity was assessed based on the relative abundance of the 18S and 28S ribosomal bands and on the presence of baseline rise, both of which reveal RNA degradation. Gene expression profiles were performed by following the protocol recommended by Affymetrix, Inc. cRNAs were hybridized/scanned on Human Genome U133 Plus 2.0 microarrays.

### Microarray Data Preprocessing

The microarray data was summarized using Robust Multi-array Average (RMA) procedure. In the cases where there were multiple probes representing a gene on the chip, we used average of the readings obtained from different probes to obtain the expression level of that gene. The microarray data for BT1+/−, BT2+/−, N1+/− and N2+/− has been deposited in GEO under accession number GSE34152.

Microarray data used to generate EMT signatures are available in the Gene Expression Omnibus (GEO) database under the accession GSE9691 and GSE24202, as detailed in Taube *et al.* [12].

### Re-clustering of TCGA GBM Samples

We used TCGA level 3 gene expression data for 373 GBM and 10 normal sample available from Affymetrix© HT_HG_U133A platforms. We filtered the data to include only gene with median absolute deviation (MAD) larger than 0.5 over all GBM samples. We used consensus clustering based on k-means to detect robust clusters in the updated set of GBM samples in TCGA. We defined and used a *quality factor* to assess cleanness of consensus matrices obtained for different number of clusters. To obtain the quality factor, we evaluate 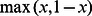 for each entry 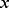 in the consensus matrix. The quality factor is defined as the average of all the above quantities obtained from entries in the consensus matrix.

### Two-sided Fisher Exact Test (TSFET)

Throughout this study, we used TSFET to evaluate significance of overlap between two subsets of genes. In the cases where gene lists were taken from different studies, we used genes whose expression levels were provided in all studies. Precisely speaking, assume the goal is to compare a gene list 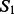 chosen from pool of genes 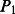 with a gene list 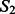 chosen from another pool of genes 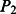. To use the TSFET, we defined the following modified sets: 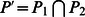, 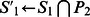 and 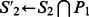. In addition, we defined 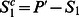 and 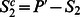, where “
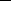
” sign denotes set difference. Next, we construct a confusion matrix, Table 8.

**Table 8:** Confusion matrix.

	(1)	(2)
**(A)**	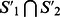 (genes in both 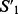 and 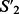 )	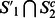 (genes in 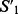 but not in 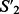 )
**(B)**	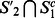 (genes in 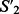 but not 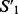 )	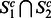 (genes in neither 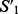 nor 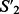 )

We use the p-value obtained by the TSFET as measure of the significance of overlap between sets 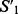 and 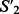. The TSFET is able to determine whether the overlap between sets 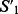 and 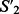 is greater than or less than what is expected due to chance. The length of overlap between 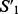 and 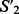 that we expected due to chance is equal to 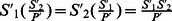.

An underlying assumption in the TSFET is that all genes in the total pool of genes in the study are likely to participate in the processes under investigation. If the set of genes that are probable to participate in the studied processes is smaller than the whole pool of genes, then the p-value obtained from TSFETs in the cases where the overlap between genetic signatures is found to be larger than what is expected due to chance is an underestimations of the true p-values. That is because TSFET assumes the genetic signatures have been chosen from an unrealistically large set of genes and yet they resulted in the observed overlap. On the other hand, in these cases, where the overlap between gene lists turns out to be less than what is expected due to chance, the p-value obtained from TSFETs is an overestimate of the true p-value. Based on this, careful attention to the background sets is required when TSFETs are used to assess significance of overlap between two signatures.

## Supporting Information

Figure S1 (A) Consensus matrices obtained from k-means and hierarchical clustering for k = to 2, 3, 4 and 5. (B) Quality factor as a function of number of clusters is plotted for consensus clustering based on hierarchical clustering. As demonstrated the quality factor has an increasing trend and does not show a drop similar to what was seen in the case of consensus clustering based on k-means.
(DOC)

Table S1 **Up and down regulated genes in the genetic signature of EMT.** (A) Up regulated genes. (B) Down regulated genes.
(DOC)

Table S2 **List of genes with similar expression in the GBM and EMT signatures.** (A) List of genes that are up regulated with a fold change of at least two in both GBM and EMT. (B) List of genes that are down regulated with a fold change of at least two in both GBM and EMT.
(DOC)

Table S3 **List of genes that are similarly or oppositely expressed in molecular subtypes of GBM and EMT signature.** The numbers are log fold change of variations in the mean of each subtype with respect to the normal samples. The GBM/Normal fold changes are evaluated separately for each GBM subtype and also for all GBM samples taken together. Dark red denotes the GBM/Normal log fold changes>1, and light red denotes a log fold change larger than 0.5 and less than 1. Similarly, dark green denotes the GBM/Normal log fold changes<−1, and light green denotes a log fold change less than −0.5 and greater than −1. (A) List of genes up-regulated in EMT and at least one of the GBM subtypes with corresponding GBM/Normal fold changes. (B) List of genes down-regulated in EMT and at least one of the GBM subtypes with corresponding GBM/Normal fold changes. (C) List of genes down-regulated in EMT and up-regulated in at least one of the GBM subtypes with corresponding GBM/Normal fold changes. (D) List of genes up-regulated in EMT and down-regulated in at least of the GBM subtypes with corresponding GBM/Normal fold changes.
(DOC)

Table S4 **List of genes that are oppositely expressed in the EMT and CD133 signatures.** (A) List of genes that are down-regulated in EMT and up-regulated in at least two out of the four CD133+ samples. (B) List of genes that are up-regulated in EMT and down-regulated in at least two out of the four CD133+ samples.
(DOC)

Table S5 **List of genes that are similarly or oppositely expressed in the genetic signatures of GBM subtypes and in the CD133 signature.** The numbers are log fold change of variations in the mean of each subtype with respect to the normal samples. Fold changes are evaluated separately for each GBM subtype and also for all GBM samples taken together. Dark red denotes the GBM/Normal log fold changes>1, and light red denotes a log fold change larger than 0.5 and less than 1. Similarly, dark green denotes the GBM/Normal log fold changes<−1, and light green denotes a log fold change less than −0.5 and greater than −1. (A) List of genes up-regulated in CD133 signature and at least one of the GBM subtypes with corresponding GBM/Normal fold changes. (B) List of genes down-regulated in CD133 signature and at least one of the GBM subtypes with corresponding GBM/Normal fold changes. (C) List of genes down-regulated in CD133 signature and up-regulated in at least one of the GBM subtypes with corresponding GBM/Normal fold changes.
(DOC)

File S1 **List of genes that are up or down-regulated with a fold change of at least two in the TCGA GBM.**
(XLS)

File S2 **List of genes that are up or down-regulated with a fold change of at least two in each of the TCGA GBM subtypes when compared to the TCGA normal samples.**
(XLS)

File S3 **List of genes that are up or down-regulated with a fold change of at least two in each of the four CD133 signatures.**
(XLS)
