# Supplementary material for: Investigating the Link between Molecular Subtypes of Glioblastoma, Epithelial-Mesenchymal Transition, and CD133 Cell Surface Protein
Source: PLoS One. 2013 May 29;8(5):e64169. doi: 10.1371/journal.pone.0064169 (PMC3667082; doi:10.1371/journal.pone.0064169)
Supplement: Figure S1 — (A) Consensus matrices obtained from k-means and hierarchical clustering for k = to 2, 3, 4 and 5. (B) Quality factor as a function of number of clusters is plotted for consensus clustering based on hierarchical clustering. As demonstrated the quality factor has an increasing trend and does not show a drop similar to what was seen in the case of consensus clustering based on k-means. (DOC) [file pone.0064169.s001.doc]

Figure S1: (A) Consensus matrices obtained from k-means and hierarchical clustering for k= to 2, 3, 4 and 5.

| K-means clustering | Hierarchial clustering |
| --- | --- |
| K=2 | K=2 |
| K=3 | K=3 |
| K=4 | K=4 |
| K=5 | K=5 |

(B) Quality factor as a function of number of clusters is plotted for consensus clustering based on hierarchical clustering. As demonstrated the quality factor has an increasing trend and does not show a drop similar to what was seen in the case of consensus clustering based on k-means**.**
