# Supplementary material for: Investigating the Link between Molecular Subtypes of Glioblastoma, Epithelial-Mesenchymal Transition, and CD133 Cell Surface Protein
Source: PLoS One. 2013 May 29;8(5):e64169. doi: 10.1371/journal.pone.0064169 (PMC3667082; doi:10.1371/journal.pone.0064169)
Supplement: Table S1 — Up and down regulated genes in the genetic signature of EMT. (A) Up regulated genes. (B) Down regulated genes. (DOC) [file pone.0064169.s002.doc]

**Table S1:** Up and down regulated genes in the genetic signature of EMT

(A) Up regulated genes

| |  | **Gene Symbol** | | --- | --- | | **1** | 'FBLN5' | | **2** | 'GREM1' | | **3** | 'COL3A1' | | **4** | 'COL1A2' | | **5** | 'DCN' | | **6** | 'CDH2' | | **7** | 'ENPP2' | | **8** | 'POSTN' | | **9** | 'RGS4' | | **10** | 'C5ORF13' | | **11** | 'PRRX1' | | **12** | 'FBN1' | | **13** | 'SRGN' | | **14** | 'SPOCK1' | | **15** | 'PRR16' | | **16** | 'DLC1' | | **17** | 'BIN1' | | **18** | 'RGL1' | | **19** | 'IGFBP4' | | **20** | 'PVRL3' | | **21** | 'CDH11' | | **22** | 'OLFML3' | | **23** | 'MMP2' | | **24** | 'MYL9' | | **25** | 'COL5A2' | | **26** | 'CTGF' | | **27** | 'PLEKHC1' | | **28** | 'ZEB1' | |  | |  | **Gene Symbol** | | --- | --- | | **29** | 'ROR1' | | **30** | 'PTGER2' | | **31** | 'CHN1' | | **32** | 'PMP22' | | **33** | 'TRAM2' | | **34** | 'TAGLN' | | **35** | 'TNFAIP6' | | **36** | 'CREB3L1' | | **37** | 'UGDH' | | **38** | 'HAS2' | | **39** | 'DNAJB4' | | **40** | 'CDKN2C' | | **41** | 'CCDC92' | | **42** | 'WNT5A' | | **43** | 'IGFBP3' | | **44** | 'PPM1D' | | **45** | 'FILIP1L' | | **46** | 'PDGFC' | | **47** | 'TBX3' | | **48** | 'DPT' | | **49** | 'STC1' | | **50** | 'LMCD1' | | **51** | 'N-PAC /// SEPT6' | | **52** | 'NR2F1' | | **53** | 'SCCPDH' | | **54** | 'MLPH' | | **55** | 'LTBP2' | | **56** | 'TPM1' | |  | |  | **Gene Symbol** | | --- | --- | | **57** | 'ANKRD25' | | **58** | 'DDR2' | | **59** | 'SEMA5A' | | **60** | 'TGFB1I1' | | **61** | 'PCOLCE' | | **62** | 'STARD13' | | **63** | 'NID1' | | **64** | 'SYNC1' | | **65** | 'ENOX1' | | **66** | 'FSTL1' | | **67** | 'VIM' | | **68** | 'MME' | | **69** | 'C10ORF56' | | **70** | 'NRP1' | | **71** | 'THY1' | | **72** | 'NEBL' | | **73** | 'TNS3' | | **74** | 'FBLN1' | | **75** | 'TUBA1A' | | **76** | 'COPZ2' | | **77** | 'CYBRD1' | | **78** | 'PPAP2B' | | **79** | 'PTX3' | | **80** | 'FADS2' | | **81** | 'BGN /// TSHZ1' | | **82** | 'ZBTB38' | |
| --- | --- | --- | --- | --- | --- | --- | --- | --- | --- | --- | --- | --- | --- | --- | --- | --- | --- | --- | --- | --- | --- | --- | --- | --- | --- | --- | --- | --- | --- | --- | --- | --- | --- | --- | --- | --- | --- | --- | --- | --- | --- | --- | --- | --- | --- | --- | --- | --- | --- | --- | --- | --- | --- | --- | --- | --- | --- | --- | --- | --- | --- | --- | --- | --- | --- | --- | --- | --- | --- | --- | --- | --- | --- | --- | --- | --- | --- | --- | --- | --- | --- | --- | --- | --- | --- | --- | --- | --- | --- | --- | --- | --- | --- | --- | --- | --- | --- | --- | --- | --- | --- | --- | --- | --- | --- | --- | --- | --- | --- | --- | --- | --- | --- | --- | --- | --- | --- | --- | --- | --- | --- | --- | --- | --- | --- | --- | --- | --- | --- | --- | --- | --- | --- | --- | --- | --- | --- | --- | --- | --- | --- | --- | --- | --- | --- | --- | --- | --- | --- | --- | --- | --- | --- | --- | --- | --- | --- | --- | --- | --- | --- | --- | --- | --- | --- | --- | --- | --- | --- | --- | --- | --- | --- | --- |

(B) Down regulated genes

| |  | **Gene Symbol** | | --- | --- | | **1** | 'C20ORF19' | | **2** | 'SNX10' | | **3** | 'TP73L' | | **4** | 'KCNK1' | | **5** | 'BDKRB2' | | **6** | 'ANXA8 /// ///ANXA8L1 /// ///LOC728113' | | **7** | 'RHBDF2' | | **8** | 'LOC653562 /// ///SLC6A10P /// ///SLC6A8' | | **9** | 'KRT18' | | **10** | 'CDS1' | | **11** | 'THBD' | | **12** | 'NEFM' | | **13** | 'RPS6KA1' | | **14** | 'SMPDL3B' | | **15** | 'ABCA12' | | **16** | 'RHOD' | | **17** | 'KRT14' | | **18** | 'PRKCH' | | **19** | 'ZBED2' | | **20** | 'C10ORF10' | | **21** | 'LRRC1' | | **22** | 'STAP2' | | **23** | 'JUP' | | **24** | 'IL4R' | | **25** | 'PERP' | | **26** | 'FGFBP1' | | **27** | 'MYO1D' | | **28** | 'FAT2' | | **29** | 'WWC1' | | **30** | 'FZD3' | | **31** | 'ZNF165' | | **32** | 'SNCA' | | **33** | 'KIAA1815' | | **34** | 'PRSS8' | | **35** | 'SH2D3A' | | **36** | 'GNAL' | | **37** | 'BIK' | | **38** | 'CDH3' | | **39** | 'KIAA0888' | | **40** | 'KRT5' | | **41** | 'GJB3' | | **42** | 'KIAA0040' | | **43** | 'CELSR2' | | **44** | 'F11R' | | **45** | 'NUP62CL' | | **46** | 'SERPINB1' | | **47** | 'SPINT2' | | **48** | 'POLR3G' | | **49** | 'ELMO3' | |  | |  | **Gene Symbol** | | --- | --- | | **50** | 'IL1RN' | | **51** | 'TSPAN1' | | **52** | 'IFI30' | | **53** | 'PLS1' | | **54** | 'LOC729884 /// ///TMPRSS11E' | | **55** | 'C1ORF116' | | **56** | 'ALOX15B' | | **57** | 'COL17A1' | | **58** | 'RTEL1 /// ///TNFRSF6B' | | **59** | 'LAD1' | | **60** | 'PTPN3' | | **61** | 'MST1R' | | **62** | 'EPHA1' | | **63** | 'E2F5' | | **64** | 'KRT6B' | | **65** | 'STAC' | | **66** | 'ITGB4' | | **67** | 'C6ORF105' | | **68** | 'GLS2' | | **69** | 'ANXA3' | | **70** | 'DST' | | **71** | 'ARHGAP25' | | **72** | 'DSC2' | | **73** | 'SLC6A8' | | **74** | 'LSR' | | **75** | 'CLDN1' | | **76** | 'FLJ20366' | | **77** | 'CYP4F11' | | **78** | 'CCND2' | | **79** | 'FGFR2' | | **80** | 'ABLIM1' | | **81** | 'XDH' | | **82** | 'CAMK2B' | | **83** | 'DSG3' | | **84** | 'NAIP /// OCLN' | | **85** | 'KRT17' | | **86** | 'SAA1 /// SAA2' | | **87** | 'PRRG4' | | **88** | 'ANK3' | | **89** | 'TMPRSS4' | | **90** | 'CST6' | | **91** | 'NDRG1' | | **92** | 'S100A8' | | **93** | 'CORO1A' | | **94** | 'KLK5' | | **95** | 'EXPH5' | | **96** | 'IRX4' | | **97** | 'IRF6' | | **98** | 'HOOK1' | | **99** | 'ARTN' | | **100** | 'FLJ12684' | |  | |  | **Gene Symbol** | | --- | --- | | **101** | 'SLC2A9' | | **102** | 'KLK8' | | **103** | 'TMEM40' | | **104** | 'TRIM29' | | **105** | 'HBEGF' | | **106** | 'ALDH1A3' | | **107** | 'RBM35B' | | **108** | 'MYO5C' | | **109** | 'CYP27B1' | | **110** | 'IL1B' | | **111** | 'NMU' | | **112** | 'KRT16' | | **113** | 'CDH1' | | **114** | 'JAG2' | | **115** | 'VSNL1' | | **116** | 'RLN2' | | **117** | 'CTSL2' | | **118** | 'SYK' | | **119** | 'SAA1' | | **120** | 'EPB41L4B' | | **121** | 'RNF128' | | **122** | 'ST14' | | **123** | 'LEPREL1' | | **124** | 'PI3' | | **125** | 'AP1M2' | | **126** | 'CKMT1A /// ///CKMT1B' | | **127** | 'GRHL2' | | **128** | 'ARHGAP8 /// ///LOC553158' | | **129** | 'IL18' | | **130** | 'CA9' | | **131** | 'S100A14' | | **132** | 'CA2' | | **133** | 'KRT15' | | **134** | 'EVA1' | | **135** | 'TMEM30B' | | **136** | 'S100A7' | | **137** | 'KLK7' | | **138** | 'LGALS7' | | **139** | 'FST' | | **140** | 'CXADR' | | **141** | 'SLPI' | | **142** | 'RBM35A' | | **143** | 'RAB25' | | **144** | 'UCHL1' | | **145** | 'KLK10' | | **146** | 'TACSTD1' | | **147** | 'SERPINB2' | | **148** | 'SPRR1A' | | **149** | 'FGFR3' | | **150** | 'SPRR1B' | |
| --- | --- | --- | --- | --- | --- | --- | --- | --- | --- | --- | --- | --- | --- | --- | --- | --- | --- | --- | --- | --- | --- | --- | --- | --- | --- | --- | --- | --- | --- | --- | --- | --- | --- | --- | --- | --- | --- | --- | --- | --- | --- | --- | --- | --- | --- | --- | --- | --- | --- | --- | --- | --- | --- | --- | --- | --- | --- | --- | --- | --- | --- | --- | --- | --- | --- | --- | --- | --- | --- | --- | --- | --- | --- | --- | --- | --- | --- | --- | --- | --- | --- | --- | --- | --- | --- | --- | --- | --- | --- | --- | --- | --- | --- | --- | --- | --- | --- | --- | --- | --- | --- | --- | --- | --- | --- | --- | --- | --- | --- | --- | --- | --- | --- | --- | --- | --- | --- | --- | --- | --- | --- | --- | --- | --- | --- | --- | --- | --- | --- | --- | --- | --- | --- | --- | --- | --- | --- | --- | --- | --- | --- | --- | --- | --- | --- | --- | --- | --- | --- | --- | --- | --- | --- | --- | --- | --- | --- | --- | --- | --- | --- | --- | --- | --- | --- | --- | --- | --- | --- | --- | --- | --- | --- | --- | --- | --- | --- | --- | --- | --- | --- | --- | --- | --- | --- | --- | --- | --- | --- | --- | --- | --- | --- | --- | --- | --- | --- | --- | --- | --- | --- | --- | --- | --- | --- | --- | --- | --- | --- | --- | --- | --- | --- | --- | --- | --- | --- | --- | --- | --- | --- | --- | --- | --- | --- | --- | --- | --- | --- | --- | --- | --- | --- | --- | --- | --- | --- | --- | --- | --- | --- | --- | --- | --- | --- | --- | --- | --- | --- | --- | --- | --- | --- | --- | --- | --- | --- | --- | --- | --- | --- | --- | --- | --- | --- | --- | --- | --- | --- | --- | --- | --- | --- | --- | --- | --- | --- | --- | --- | --- | --- | --- | --- | --- | --- | --- | --- | --- | --- | --- | --- | --- | --- | --- | --- | --- | --- | --- | --- | --- | --- | --- | --- | --- | --- | --- | --- | --- | --- | --- |
