# Supplementary material for: Investigating the Link between Molecular Subtypes of Glioblastoma, Epithelial-Mesenchymal Transition, and CD133 Cell Surface Protein
Source: PLoS One. 2013 May 29;8(5):e64169. doi: 10.1371/journal.pone.0064169 (PMC3667082; doi:10.1371/journal.pone.0064169)
Supplement: Table S2 — List of genes with similar expression in the GBM and EMT signatures. (A) List of genes that are up regulated with a fold change of at least two in both GBM and EMT. (B) List of genes that are down regulated with a fold change of at least two in both GBM and EMT. (DOC) [file pone.0064169.s003.doc]

**Table S2:** List of genes with similar expression in the GBM and EMT signatures.

(A) List of genes that are up regulated with a fold change of at least two in both GBM and EMT.

|  | **Gene symbol** | **Log2 of FC in GBM/Normal** |
| --- | --- | --- |
| **1** | 'POSTN' | 3.92 |
| **2** | 'PTX3' | 3.81 |
| **3** | 'TGFB1I1' | 3.35 |
| **4** | 'COL3A1' | 3.32 |
| **5** | 'IGFBP3' | 3.11 |
| **6** | 'COL5A2' | 2.89 |
| **7** | 'FSTL1' | 2.70 |
| **8** | 'MMP2' | 2.62 |
| **9** | 'COL1A2' | 2.57 |
| **10** | 'CDKN2C' | 2.54 |
| **11** | 'NID1' | 2.53 |
| **12** | 'TAGLN' | 2.35 |
| **13** | 'TNFAIP6' | 2.27 |
| **14** | 'CYBRD1' | 2.07 |
| **15** | 'VIM' | 2.01 |
| **16** | 'WNT5A' | 1.96 |
| **17** | 'PCOLCE' | 1.89 |
| **18** | 'IGFBP4' | 1.77 |
| **19** | 'PDGFC' | 1.73 |
| **20** | 'CDH11' | 1.72 |
| **21** | 'OLFML3' | 1.70 |
| **22** | 'PRRX1' | 1.67 |
| **23** | 'UGDH' | 1.61 |
| **24** | 'SRGN' | 1.60 |
| **25** | 'ANKRD25' | 1.50 |
| **26** | 'COPZ2' | 1.43 |
| **27** | 'SEMA5A' | 1.30 |
| **28** | 'PPM1D' | 1.25 |
| **29** | 'DDR2' | 1.25 |
| **30** | 'TRAM2' | 1.20 |
| **31** | 'HAS2' | 1.19 |
| **32** | 'SYNC1' | 1.18 |
| **33** | 'FILIP1L' | 1.11 |
| **34** | 'CTGF' | 1.04 |

(B) List of genes that are down regulated with a fold change of at least two in both GBM and EMT.

|  | **Gene symbol** | **Log2 of FC in GBM/Normal** |
| --- | --- | --- |
| **1** | 'VSNL1' | -6.18 |
| **2** | 'NEFM' | -5.59 |
| **3** | 'KCNK1' | -4.52 |
| **4** | 'SNCA' | -4.38 |
| **5** | 'CAMK2B' | -3.95 |
| **6** | 'ANK3' | -2.87 |
| **7** | 'SPINT2' | -2.79 |
| **8** | 'GLS2' | -2.49 |
| **9** | 'UCHL1' | -2.20 |
| **10** | 'KLK7' | -2.11 |
| **11** | 'TACSTD1' | -1.70 |
| **12** | 'KRT17' | -1.54 |
| **13** | 'ANXA3' | -1.49 |
| **14** | 'PTPN3' | -1.41 |
| **15** | 'KIAA0888' | -1.30 |
| **16** | 'FGFR3' | -1.22 |
| **17** | 'SNX10' | -1.17 |
| **18** | 'CELSR2' | -1.15 |
| **19** | 'GNAL' | -1.12 |
| **20** | 'RNF128' | -1.03 |
| **21** | 'HOOK1' | -1.01 |
| **22** | 'JAG2' | -1.01 |
