# Supplementary material for: Investigating the Link between Molecular Subtypes of Glioblastoma, Epithelial-Mesenchymal Transition, and CD133 Cell Surface Protein
Source: PLoS One. 2013 May 29;8(5):e64169. doi: 10.1371/journal.pone.0064169 (PMC3667082; doi:10.1371/journal.pone.0064169)
Supplement: Table S4 — List of genes that are oppositely expressed in the EMT and CD133 signatures. (A) List of genes that are down-regulated in EMT and up-regulated in at least two out of the four CD133+ samples. (B) List of genes that are up-regulated in EMT and down-regulated in at least two out of the four CD133+ samples. (DOC) [file pone.0064169.s005.doc]

**Table S4:** List of genes that are oppositely expressed in the EMT and CD133 signatures.

(A) List of genes that are down-regulated in EMT and up-regulated in at least two out of the four CD133+ samples.

|  | **Gene symbol** | **No. of occurrence** |
| --- | --- | --- |
| **1** | 'IL18' | 4 |
| **2** | 'KRT14' | 3 |
| **3** | 'F11R' | 3 |
| **4** | 'IL1RN' | 3 |
| **5** | 'ALOX15B' | 3 |
| **6** | 'ARHGAP25' | 3 |
| **7** | 'CST6' | 3 |
| **8** | 'SYK' | 3 |
| **9** | 'ST14' | 3 |
| **10** | 'SNX10' | 2 |
| **11** | 'KRT18' | 2 |
| **12** | 'CDS1' | 2 |
| **13** | 'THBD' | 2 |
| **14** | 'ZBED2' | 2 |
| **15** | 'FGFBP1' | 2 |
| **16** | 'SNCA' | 2 |
| **17** | 'PRSS8' | 2 |
| **18** | 'BIK' | 2 |
| **19** | 'TSPAN1' | 2 |
| **20** | 'PLS1' | 2 |
| **21** | 'MST1R' | 2 |
| **22** | 'KRT6B' | 2 |
| **23** | 'GLS2' | 2 |
| **24** | 'CLDN1' | 2 |
| **25** | 'CYP4F11' | 2 |
| **26** | 'SAA1 /// SAA2' | 2 |
| **27** | 'TMPRSS4' | 2 |
| **28** | 'S100A8' | 2 |
| **29** | 'IRF6' | 2 |
| **30** | 'HOOK1' | 2 |
| **31** | 'IL1B' | 2 |
| **32** | 'CDH1' | 2 |
| **33** | 'JAG2' | 2 |
| **34** | 'CTSL2' | 2 |
| **35** | 'PI3' | 2 |
| **36** | 'AP1M2' | 2 |
| **37** | 'GRHL2' | 2 |
| **38** | 'CA2' | 2 |
| **39** | 'KLK7' | 2 |
| **40** | 'SLPI' | 2 |
| **41** | 'RBM35A' | 2 |
| **42** | 'RAB25' | 2 |
| **43** | 'KLK10' | 2 |
| **44** | 'TACSTD1' | 2 |

(B) List of genes that are up-regulated in EMT and down-regulated in at least two out of the four CD133+ samples.

|  | **Gene symbol** | **No. of occurrence** |
| --- | --- | --- |
| **1** | 'FBLN5' | 2 |
| **2** | 'ENPP2' | 2 |
| **3** | 'SPOCK1' | 2 |
| **4** | 'PRR16' | 2 |
| **5** | 'COL5A2' | 2 |
| **6** | 'ENOX1' | 2 |
| **7** | 'PTX3' | 2 |
