# Supplementary material for: Investigating the Link between Molecular Subtypes of Glioblastoma, Epithelial-Mesenchymal Transition, and CD133 Cell Surface Protein
Source: PLoS One. 2013 May 29;8(5):e64169. doi: 10.1371/journal.pone.0064169 (PMC3667082; doi:10.1371/journal.pone.0064169)
Supplement: Table S5 — List of genes that are similarly or oppositely expressed in the genetic signatures of GBM subtypes and in the CD133 signature. The numbers are log fold change of variations in the mean of each subtype with respect to the normal samples. Fold changes are evaluated separately for each GBM subtype and also for all GBM samples taken together. Dark red denotes the GBM/Normal log fold changes>1, and light red denotes a log fold change larger than 0.5 and less than 1. Similarly, dark green denotes the GBM/Normal log fold changes<−1, and light green denotes a log fold change less than −0.5 and greater than −1. (A) List of genes up-regulated in CD133 signature and at least one of the GBM subtypes with corresponding GBM/Normal fold changes. (B) List of genes down-regulated in CD133 signature and at least one of the GBM subtypes with corresponding GBM/Normal fold changes. (C) List of genes down-regulated in CD133 signature and up-regulated in at least one of the GBM subtypes with corresponding GBM/Normal fold changes. (DOC) [file pone.0064169.s006.doc]

**Table S5:** List of genes that are similarly or oppositely expressed in the genetic signatures of GBM subtypes and in the CD133 signature. The numbers are log fold change of variations in the mean of each subtype with respect to the normal samples. Fold changes are evaluated separately for each GBM subtype and also for all GBM samples taken together. Dark red denotes the GBM/Normal log fold changes>1, and light red denotes a log fold change larger than 0.5 and less than 1. Similarly, dark green denotes the GBM/Normal log fold changes<-1, and light green denotes a log fold change less than -0.5 and greater than -1.

(A) List of genes up-regulated in CD133 signature and at least one of the GBM subtypes with corresponding GBM/Normal fold changes.

|  | **Gene symbol** | **Mesenchymal** | **Classical** | **Neural** | **Proneural** | **All GBM samples** |
| --- | --- | --- | --- | --- | --- | --- |
| **1** | 'RRM2' | 4.16 | 4.00 | 2.80 | 4.60 | 3.94 |
| **2** | 'CHI3L2' | 4.69 | 3.49 | 3.84 | 2.83 | 3.79 |
| **3** | 'CD163' | 5.15 | 3.02 | 2.99 | 2.93 | 3.67 |
| **4** | 'MS4A6A' | 4.12 | 2.68 | 3.56 | 2.62 | 3.30 |
| **5** | 'LPL' | 3.15 | 3.67 | 2.99 | 2.97 | 3.21 |
| **6** | 'MS4A4A' | 4.39 | 2.25 | 3.17 | 2.66 | 3.20 |
| **7** | 'VSIG4' | 4.05 | 2.14 | 3.10 | 2.37 | 2.99 |
| **8** | 'CPVL' | 3.69 | 2.98 | 2.89 | 2.02 | 2.97 |
| **9** | 'FAM70A' | 2.73 | 2.92 | 2.94 | 3.17 | 2.92 |
| **10** | 'LYZ' | 3.91 | 2.32 | 2.75 | 2.21 | 2.89 |
| **11** | 'CD93' | 3.54 | 2.68 | 2.12 | 2.56 | 2.81 |
| **12** | 'APOC1' | 3.12 | 2.29 | 3.15 | 2.48 | 2.77 |
| **13** | 'SOD2' | 3.67 | 2.47 | 2.28 | 1.96 | 2.70 |
| **14** | 'CD14' | 3.76 | 2.00 | 2.53 | 2.03 | 2.67 |
| **15** | 'C1QA' | 3.56 | 1.87 | 2.80 | 2.10 | 2.64 |
| **16** | 'C1QB' | 3.35 | 1.75 | 2.70 | 2.05 | 2.51 |
| **17** | 'CXCR4' | 3.18 | 2.38 | 2.21 | 1.92 | 2.50 |
| **18** | 'FCER1G' | 3.42 | 1.87 | 2.54 | 1.87 | 2.50 |
| **19** | 'HLA-DMA' | 3.26 | 1.96 | 2.77 | 1.75 | 2.49 |
| **20** | 'TREM2' | 2.97 | 1.95 | 2.93 | 1.83 | 2.44 |
| **21** | 'HLA-DRA' | 3.10 | 1.84 | 2.59 | 1.64 | 2.35 |
| **22** | 'SERPINA1' | 3.56 | 1.71 | 2.18 | 1.49 | 2.34 |
| **23** | 'STAB1' | 3.24 | 1.73 | 1.96 | 1.75 | 2.26 |
| **24** | 'SAMSN1' | 2.99 | 1.51 | 2.46 | 1.66 | 2.20 |
| **25** | 'VAMP8' | 3.09 | 1.55 | 2.32 | 1.55 | 2.19 |
| **26** | 'TSPAN12' | 1.47 | 2.55 | 2.54 | 2.48 | 2.19 |
| **27** | 'ITGB2' | 2.98 | 1.50 | 2.24 | 1.34 | 2.08 |
| **28** | 'SLC7A7' | 2.72 | 1.63 | 2.23 | 1.53 | 2.07 |
| **29** | 'RNASE6' | 2.88 | 1.51 | 2.29 | 1.39 | 2.07 |
| **30** | 'CSTA' | 3.10 | 1.54 | 1.92 | 1.12 | 2.01 |
| **31** | 'CTSS' | 2.74 | 1.35 | 2.12 | 1.42 | 1.96 |
| **32** | 'NUPR1' | 2.50 | 1.65 | 2.00 | 1.51 | 1.96 |
| **33** | 'NPL' | 2.54 | 1.44 | 2.22 | 1.42 | 1.94 |
| **34** | 'HLA-DPA1' | 2.53 | 1.48 | 2.18 | 1.36 | 1.93 |
| **35** | 'TLR2' | 2.79 | 1.41 | 1.97 | 1.18 | 1.91 |
| **36** | 'TYROBP' | 2.62 | 1.31 | 2.23 | 1.27 | 1.90 |
| **37** | 'RNASE4' | 2.46 | 1.96 | 1.50 | 1.13 | 1.84 |
| **38** | 'SQRDL' | 2.63 | 1.45 | 1.81 | 1.05 | 1.81 |
| **39** | 'DRAM' | 2.38 | 1.92 | 1.44 | 1.14 | 1.80 |
| **40** | 'CD74' | 2.39 | 1.39 | 2.06 | 1.15 | 1.79 |
| **41** | 'TREM1' | 3.05 | 1.24 | 1.08 | 1.27 | 1.79 |
| **42** | 'ADORA3' | 2.31 | 1.30 | 2.21 | 1.19 | 1.78 |
| **43** | 'CP' | 2.84 | 1.21 | 1.64 | 1.04 | 1.77 |
| **44** | 'ARHGDIB' | 2.30 | 1.38 | 1.88 | 1.36 | 1.77 |
| **45** | 'LAPTM5' | 2.53 | 1.23 | 1.86 | 1.19 | 1.76 |
| **46** | 'INSM1' | 1.45 | 1.18 | 1.19 | 3.40 | 1.75 |
| **47** | 'HAMP' | 2.71 | 1.14 | 1.95 | 0.91 | 1.75 |
| **48** | 'HLA-DMB' | 2.32 | 1.21 | 2.15 | 1.12 | 1.73 |
| **49** | 'ALOX5AP' | 2.70 | 1.37 | 1.66 | 0.81 | 1.73 |
| **50** | 'PROM1' | 1.05 | 1.62 | 1.83 | 2.70 | 1.72 |
| **51** | 'C5AR1' | 2.70 | 1.31 | 1.35 | 1.10 | 1.71 |
| **52** | 'GPR65' | 2.34 | 1.29 | 1.86 | 1.18 | 1.71 |
| **53** | 'CLEC5A' | 2.59 | 1.82 | 1.08 | 0.88 | 1.71 |
| **54** | 'HCLS1' | 2.52 | 1.12 | 1.94 | 0.98 | 1.70 |
| **55** | 'C3' | 2.37 | 1.36 | 2.02 | 0.80 | 1.69 |
| **56** | 'PTPRC' | 2.45 | 1.14 | 1.88 | 0.95 | 1.66 |
| **57** | 'EVI2B' | 2.29 | 1.05 | 2.16 | 0.88 | 1.63 |
| **58** | 'PIPOX' | 1.60 | 2.79 | 1.70 | 0.13 | 1.61 |
| **59** | 'SRGN' | 2.42 | 1.10 | 1.51 | 1.10 | 1.60 |
| **60** | 'SCIN' | 2.10 | 1.01 | 1.86 | 1.33 | 1.60 |
| **61** | 'HLA-DRB1' | 2.26 | 1.13 | 1.78 | 1.01 | 1.59 |
| **62** | 'TMEM140' | 1.98 | 1.49 | 1.68 | 0.93 | 1.56 |
| **63** | 'TMEM176A' | 2.15 | 1.13 | 1.82 | 0.91 | 1.54 |
| **64** | 'GPX3' | 2.02 | 1.31 | 1.23 | 1.13 | 1.48 |
| **65** | 'POU3F2' | 1.22 | 2.07 | 1.23 | 1.37 | 1.48 |
| **66** | 'PLAC8' | 1.68 | 1.43 | 1.86 | 0.90 | 1.48 |
| **67** | 'IL10RA' | 2.35 | 0.81 | 1.66 | 0.81 | 1.47 |
| **68** | 'FCGR1A' | 2.12 | 0.93 | 1.79 | 0.87 | 1.46 |
| **69** | 'S100A8' | 2.56 | 0.94 | 1.31 | 0.64 | 1.46 |
| **70** | 'APOC2' | 1.93 | 0.90 | 1.96 | 0.95 | 1.45 |
| **71** | 'CCL2' | 2.86 | 0.93 | 1.29 | 0.19 | 1.45 |
| **72** | 'LYN' | 2.04 | 1.23 | 1.54 | 0.76 | 1.45 |
| **73** | 'BCL2A1' | 2.30 | 0.81 | 1.28 | 1.01 | 1.42 |
| **74** | 'UCP2' | 1.77 | 0.73 | 1.61 | 1.51 | 1.41 |
| **75** | 'GMFG' | 2.02 | 1.00 | 1.52 | 0.85 | 1.39 |
| **76** | 'GYPC' | 2.00 | 0.74 | 1.48 | 1.06 | 1.36 |
| **77** | 'CEP55' | 1.60 | 1.13 | 0.74 | 1.80 | 1.35 |
| **78** | 'HCK' | 2.17 | 0.74 | 1.52 | 0.70 | 1.34 |
| **79** | 'TMEM149' | 1.51 | 1.30 | 1.44 | 1.02 | 1.34 |
| **80** | 'CCR1' | 2.03 | 0.88 | 1.46 | 0.76 | 1.33 |
| **81** | 'FCGR2B' | 2.66 | 0.86 | 0.78 | 0.47 | 1.33 |
| **82** | 'ITGA7' | 1.47 | 2.07 | 0.87 | 0.59 | 1.31 |
| **83** | 'RTP4' | 1.55 | 1.24 | 1.51 | 0.79 | 1.29 |
| **84** | 'CENTA2' | 1.84 | 0.84 | 1.42 | 0.92 | 1.29 |
| **85** | 'S100A9' | 2.28 | 0.85 | 0.96 | 0.67 | 1.28 |
| **86** | 'tcag7.1314' | 1.85 | 1.47 | 1.10 | 0.40 | 1.28 |
| **87** | 'HPSE' | 1.74 | 0.72 | 1.36 | 1.15 | 1.27 |
| **88** | 'VWF' | 1.36 | 1.47 | 1.04 | 1.08 | 1.26 |
| **89** | 'LEFTY2' | 1.50 | 0.95 | 1.40 | 1.04 | 1.23 |
| **90** | 'MGAT4A' | 1.71 | 0.91 | 1.27 | 0.88 | 1.23 |
| **91** | 'HCP5' | 1.62 | 1.07 | 1.44 | 0.54 | 1.20 |
| **92** | 'NCF2' | 1.90 | 0.76 | 1.16 | 0.75 | 1.20 |
| **93** | 'PSCDBP' | 1.92 | 0.85 | 1.01 | 0.72 | 1.19 |
| **94** | 'ADAMDEC1' | 1.48 | 0.80 | 1.07 | 1.35 | 1.19 |
| **95** | 'CYBB' | 1.85 | 0.84 | 1.20 | 0.63 | 1.19 |
| **96** | 'MYO1F' | 1.78 | 0.77 | 1.26 | 0.67 | 1.17 |
| **97** | 'IGSF6' | 1.42 | 0.75 | 1.60 | 0.87 | 1.16 |
| **98** | 'CLEC7A' | 1.79 | 0.79 | 1.38 | 0.43 | 1.15 |
| **99** | 'GIMAP6' | 1.68 | 0.80 | 1.33 | 0.62 | 1.15 |
| **100** | 'C3AR1' | 1.88 | 0.55 | 1.41 | 0.48 | 1.13 |
| **101** | 'CA2' | 1.12 | 1.47 | 1.28 | 0.58 | 1.13 |
| **102** | 'PGDS' | 1.57 | 0.57 | 1.74 | 0.59 | 1.12 |
| **103** | 'LY75' | 1.89 | 0.73 | 1.17 | 0.41 | 1.11 |
| **104** | 'SLC2A5' | 1.82 | 0.66 | 1.20 | 0.56 | 1.11 |
| **105** | 'C2' | 1.91 | 0.49 | 1.15 | 0.67 | 1.11 |
| **106** | 'PDLIM3' | 1.28 | 1.03 | 1.05 | 0.96 | 1.10 |
| **107** | 'NINJ1' | 1.35 | 0.71 | 1.29 | 0.93 | 1.08 |
| **108** | 'LCP2' | 1.75 | 0.67 | 1.11 | 0.56 | 1.07 |
| **109** | 'SYK' | 1.67 | 0.57 | 1.23 | 0.62 | 1.06 |
| **110** | 'CSF1R' | 1.79 | 0.47 | 1.46 | 0.33 | 1.06 |
| **111** | 'TNFRSF1B' | 1.80 | 0.62 | 1.02 | 0.49 | 1.05 |
| **112** | 'CD86' | 1.61 | 0.63 | 1.29 | 0.48 | 1.04 |
| **113** | 'CD48' | 1.50 | 0.76 | 1.16 | 0.59 | 1.04 |
| **114** | 'WSCD1' | 0.34 | 1.88 | 0.87 | 1.18 | 1.04 |
| **115** | 'PODXL' | 1.30 | 1.11 | 0.63 | 0.92 | 1.03 |
| **116** | 'PI3' | 2.21 | 0.61 | 0.27 | 0.46 | 1.02 |
| **117** | 'LST1' | 1.44 | 0.60 | 1.38 | 0.56 | 1.01 |
| **118** | 'SLAMF8' | 1.77 | 0.52 | 0.86 | 0.63 | 1.01 |
| **119** | 'TFEC' | 1.47 | 0.57 | 1.25 | 0.59 | 0.99 |
| **120** | 'NCKAP1L' | 1.60 | 0.58 | 1.07 | 0.52 | 0.99 |
| **121** | 'LY86' | 1.57 | 0.36 | 1.67 | 0.26 | 0.98 |
| **122** | 'MSTN' | 0.43 | 0.69 | 1.72 | 1.43 | 0.98 |
| **123** | 'DENND2D' | 1.55 | 0.52 | 1.11 | 0.57 | 0.98 |
| **124** | 'SLA' | 1.81 | 0.40 | 1.10 | 0.35 | 0.98 |
| **125** | 'HOXB7' | 1.23 | 1.06 | 0.93 | 0.55 | 0.98 |
| **126** | 'EBI2' | 1.93 | 0.47 | 0.83 | 0.33 | 0.97 |
| **127** | 'ARHGAP15' | 1.38 | 0.64 | 1.20 | 0.56 | 0.97 |
| **128** | 'C1orf38' | 1.76 | 0.42 | 1.00 | 0.42 | 0.96 |
| **129** | 'C1orf106' | 0.49 | 0.75 | 0.78 | 2.02 | 0.95 |
| **130** | 'LAIR1' | 1.60 | 0.54 | 0.97 | 0.44 | 0.94 |
| **131** | 'FGL2' | 1.53 | 0.55 | 1.13 | 0.35 | 0.93 |
| **132** | 'CD37' | 1.41 | 0.47 | 1.28 | 0.46 | 0.93 |
| **133** | 'SPC25' | 0.76 | 0.87 | 0.45 | 1.68 | 0.93 |
| **134** | 'FYB' | 1.48 | 0.45 | 1.17 | 0.47 | 0.93 |
| **135** | 'GIMAP4' | 1.57 | 0.48 | 1.26 | 0.21 | 0.92 |
| **136** | 'IL18' | 1.34 | 0.54 | 1.32 | 0.38 | 0.91 |
| **137** | 'LILRB1' | 1.55 | 0.45 | 0.98 | 0.38 | 0.89 |
| **138** | 'TBXAS1' | 1.40 | 0.49 | 1.11 | 0.35 | 0.87 |
| **139** | 'LXN' | 1.03 | 1.06 | 0.76 | 0.53 | 0.87 |
| **140** | 'CXorf21' | 1.06 | 0.66 | 0.96 | 0.75 | 0.87 |
| **141** | 'ENTPD1' | 1.09 | 0.69 | 0.86 | 0.65 | 0.84 |
| **142** | 'PCDH12' | 1.01 | 0.87 | 0.57 | 0.74 | 0.83 |
| **143** | 'SYNGR2' | 1.41 | 0.27 | 1.10 | 0.41 | 0.83 |
| **144** | 'PROCR' | 1.24 | 0.64 | 0.78 | 0.50 | 0.83 |
| **145** | 'F11R' | 1.20 | 0.86 | 0.67 | 0.36 | 0.82 |
| **146** | 'SLC15A3' | 1.24 | 0.57 | 0.84 | 0.43 | 0.80 |
| **147** | 'CECR1' | 1.34 | 0.55 | 1.10 | 0.05 | 0.80 |
| **148** | 'BLNK' | 1.07 | 0.49 | 1.56 | 0.05 | 0.79 |
| **149** | 'TLR5' | 1.26 | 0.31 | 1.23 | 0.27 | 0.79 |
| **150** | 'TLR7' | 1.30 | 0.35 | 1.25 | 0.13 | 0.78 |
| **151** | 'CD38' | 0.74 | 0.46 | 1.30 | 0.67 | 0.77 |
| **152** | 'THBD' | 1.57 | 0.37 | 0.36 | 0.40 | 0.76 |
| **153** | 'PGCP' | 1.17 | 0.80 | 0.73 | 0.11 | 0.75 |
| **154** | 'NCF4' | 1.32 | 0.38 | 0.81 | 0.33 | 0.75 |
| **155** | 'AIF1' | 1.39 | 0.22 | 1.04 | 0.17 | 0.75 |
| **156** | 'CFD' | 1.40 | 0.16 | 0.92 | 0.32 | 0.74 |
| **157** | 'LCP1' | 1.27 | 0.34 | 0.74 | 0.43 | 0.74 |
| **158** | 'P2RX4' | 1.07 | 0.62 | 0.63 | 0.48 | 0.73 |
| **159** | 'FCGR3B' | 1.23 | 0.38 | 0.73 | 0.41 | 0.73 |
| **160** | 'SLC11A1' | 1.21 | 0.44 | 0.64 | 0.39 | 0.71 |
| **161** | 'IL1R2' | 1.47 | 0.23 | 0.33 | 0.53 | 0.71 |
| **162** | 'FGR' | 1.18 | 0.41 | 0.65 | 0.42 | 0.71 |
| **163** | 'CSF2RB' | 1.28 | 0.31 | 0.78 | 0.28 | 0.70 |
| **164** | 'OLR1' | 1.05 | 0.32 | 1.16 | 0.23 | 0.70 |
| **165** | 'CD300A' | 1.05 | 0.39 | 0.65 | 0.50 | 0.67 |
| **166** | 'OGFRL1' | 1.04 | 0.45 | 0.95 | 0.07 | 0.65 |
| **167** | 'ICAM1' | 1.36 | 0.51 | 0.28 | 0.15 | 0.65 |
| **168** | 'NOD2' | 1.08 | 0.41 | 0.51 | 0.33 | 0.62 |
| **169** | 'SLC22A18' | 1.06 | 0.94 | 0.26 | -0.05 | 0.62 |
| **170** | 'SLCO2B1' | 1.07 | 0.30 | 0.71 | 0.27 | 0.62 |
| **171** | 'VNN2' | 1.06 | 0.32 | 0.65 | 0.23 | 0.60 |
| **172** | 'CTSD' | 1.03 | 0.35 | 0.56 | 0.31 | 0.60 |
| **173** | 'GPRC5A' | 1.17 | 0.53 | 0.18 | 0.23 | 0.60 |
| **174** | 'PTPN6' | 1.10 | 0.20 | 0.77 | 0.14 | 0.59 |
| **175** | 'ACP5' | 1.03 | 0.28 | 0.44 | 0.46 | 0.59 |
| **176** | 'KCNJ16' | -0.42 | 1.29 | 1.44 | 0.38 | 0.58 |
| **177** | 'PLEK' | 1.11 | 0.14 | 0.70 | 0.23 | 0.58 |
| **178** | 'CDC6' | 0.47 | 0.52 | 0.24 | 1.05 | 0.57 |
| **179** | 'FUCA1' | 1.15 | 0.21 | 0.62 | 0.04 | 0.55 |
| **180** | 'FOLR2' | 1.26 | -0.33 | 0.78 | 0.28 | 0.53 |
| **181** | 'AQP9' | 1.29 | -0.20 | 0.19 | 0.08 | 0.41 |
| **182** | 'CCL18' | 1.06 | -0.11 | 0.18 | 0.27 | 0.40 |
| **183** | 'MMP7' | 1.52 | -0.09 | -0.26 | -0.02 | 0.40 |
| **184** | 'KCNN3' | -0.13 | 0.05 | 1.22 | 0.26 | 0.27 |
| **185** | 'C9orf61' | -0.57 | -0.06 | 1.11 | -0.53 | -0.09 |

(B) List of genes down-regulated in CD133 signature and at least one of the GBM subtypes with corresponding GBM/Normal fold changes.

|  | **Gene symbol** | **Mesenchymal** | **Classical** | **Neural** | **Proneural** | **All GBM samples** |
| --- | --- | --- | --- | --- | --- | --- |
| **1** | 'VSNL1' | -6.47 | -6.73 | -4.96 | -6.23 | -6.18 |
| **2** | 'NEFL' | -5.72 | -5.93 | -4.21 | -4.68 | -5.24 |
| **3** | 'AK5' | -5.55 | -5.54 | -4.11 | -5.09 | -5.16 |
| **4** | 'SERPINI1' | -4.85 | -5.11 | -3.37 | -4.60 | -4.56 |
| **5** | 'STX1A' | -4.13 | -4.17 | -3.69 | -3.95 | -4.01 |
| **6** | 'NAP1L2' | -4.34 | -4.30 | -2.73 | -3.53 | -3.83 |
| **7** | 'GABRB1' | -4.36 | -3.87 | -2.46 | -3.95 | -3.76 |
| **8** | 'ZNF365' | -3.93 | -4.01 | -2.90 | -3.45 | -3.64 |
| **9** | 'NPTX2' | -3.22 | -3.62 | -2.96 | -3.44 | -3.32 |
| **10** | 'PRSS3' | -3.21 | -3.14 | -2.73 | -3.25 | -3.11 |
| **11** | 'SLC6A15' | -2.96 | -3.16 | -2.66 | -2.89 | -2.94 |
| **12** | 'ANK3' | -3.16 | -3.06 | -2.42 | -2.63 | -2.87 |
| **13** | 'ATP8A2' | -2.90 | -2.93 | -2.70 | -2.87 | -2.86 |
| **14** | 'PPFIA2' | -2.78 | -3.16 | -2.41 | -2.64 | -2.78 |
| **15** | 'SPOCK1' | -2.67 | -3.98 | -1.93 | -2.10 | -2.74 |
| **16** | 'PAK6' | -2.77 | -2.79 | -2.51 | -2.76 | -2.72 |
| **17** | 'BDNF' | -2.68 | -2.43 | -2.81 | -2.99 | -2.71 |
| **18** | 'GPC5' | -2.76 | -2.67 | -1.70 | -2.85 | -2.54 |
| **19** | 'BEGAIN' | -2.74 | -2.41 | -2.36 | -2.41 | -2.50 |
| **20** | 'PAK7' | -2.85 | -2.89 | -2.17 | -1.50 | -2.43 |
| **21** | 'ENPP2' | -1.98 | -3.85 | -1.42 | -2.28 | -2.42 |
| **22** | 'TMEM35' | -2.90 | -2.70 | -1.61 | -1.22 | -2.22 |
| **23** | 'FAM130A2' | -2.64 | -2.23 | -1.71 | -1.78 | -2.16 |
| **24** | 'DCLK1' | -2.47 | -1.88 | -1.50 | -2.40 | -2.10 |
| **25** | 'CNTN1' | -2.34 | -2.35 | -1.39 | -0.80 | -1.81 |
| **26** | 'LARP6' | -1.64 | -2.05 | -1.44 | -1.73 | -1.73 |
| **27** | 'KCNN1' | -1.74 | -1.67 | -1.37 | -1.64 | -1.62 |
| **28** | 'PENK' | -1.70 | -1.87 | -1.32 | -1.47 | -1.62 |
| **29** | 'SH3BP5' | -1.06 | -1.97 | -1.59 | -1.65 | -1.54 |
| **30** | 'GRIA3' | -2.03 | -0.99 | -1.22 | -1.09 | -1.39 |
| **31** | 'FLRT3' | -1.85 | -1.10 | -1.59 | -0.76 | -1.36 |
| **32** | 'SLC1A6' | -1.38 | -1.41 | -1.25 | -1.20 | -1.32 |
| **33** | 'RPRM' | -1.85 | -1.76 | -0.75 | -0.43 | -1.29 |
| **34** | 'NR4A2' | -1.14 | -1.59 | -1.14 | -1.14 | -1.26 |
| **35** | 'BCAS1' | -1.34 | -2.26 | -0.26 | -0.11 | -1.10 |
| **36** | 'SCG2' | -0.58 | -0.74 | -1.34 | -1.61 | -1.00 |
| **37** | 'MN1' | -1.13 | -1.07 | -0.40 | -0.39 | -0.81 |
| **38** | 'DKFZP586H2123' | -0.10 | -0.71 | -0.41 | -1.50 | -0.63 |

(C) List of genes down-regulated in CD133 signature and up-regulated in at least one of the GBM subtypes with corresponding GBM/Normal fold changes.

|  | **Gene symbol** | **Mesenchymal** | **Classical** | **Neural** | **Proneural** | **All GBM samples** |
| --- | --- | --- | --- | --- | --- | --- |
| **1** | 'PTX3' | 5.21 | 4.20 | 2.36 | 2.66 | 3.81 |
| **2** | 'EMP1' | 3.91 | 3.83 | 2.97 | 2.62 | 3.42 |
| **3** | 'COL5A2' | 3.87 | 2.97 | 1.81 | 2.37 | 2.89 |
| **4** | 'TCF12' | 2.35 | 2.98 | 2.77 | 3.15 | 2.78 |
| **5** | 'PLA2G2A' | 3.38 | 2.37 | 1.57 | 1.24 | 2.28 |
| **6** | 'MXRA5' | 3.01 | 1.34 | 1.50 | 1.65 | 1.97 |
| **7** | 'ANGPTL2' | 1.77 | 1.55 | 1.45 | 2.09 | 1.72 |
| **8** | 'IL13RA2' | 2.10 | 1.00 | 1.89 | 1.57 | 1.65 |
| **9** | 'KCNE4' | 1.94 | 1.73 | 0.87 | 0.91 | 1.44 |
| **10** | 'C17orf42' | 1.13 | 1.29 | 1.33 | 1.45 | 1.28 |
| **11** | 'CREB5' | 0.79 | 1.69 | 0.96 | 1.41 | 1.20 |
| **12** | 'DKK1' | 2.67 | 0.31 | 0.65 | 0.35 | 1.13 |
| **13** | 'ZNF302' | 0.84 | 1.39 | 1.22 | 1.11 | 1.12 |
| **14** | 'ADAMTS5' | 1.21 | 1.11 | 0.96 | 1.12 | 1.11 |
| **15** | 'SELL' | 1.60 | 0.32 | 1.90 | 0.55 | 1.09 |
| **16** | 'HOXB2' | 1.31 | 1.04 | 0.84 | 1.02 | 1.08 |
| **17** | 'PCAF' | 0.55 | 1.31 | 1.49 | 0.85 | 1.01 |
| **18** | 'HOXA1' | 1.08 | 1.03 | 1.03 | 0.79 | 0.99 |
| **19** | 'TTC27' | 1.02 | 1.00 | 0.71 | 1.07 | 0.96 |
| **20** | 'C14orf104' | 0.57 | 0.91 | 0.83 | 1.14 | 0.84 |
| **21** | 'FBLN5' | 1.23 | 0.93 | 0.56 | 0.23 | 0.80 |
| **22** | 'NOVA1' | 0.11 | 0.87 | 0.51 | 1.18 | 0.63 |
| **23** | 'CCL5' | 1.00 | 0.26 | 0.60 | 0.22 | 0.56 |
| **24** | 'GRB10' | 0.99 | 1.05 | -0.18 | -0.02 | 0.55 |
| **25** | 'KLHL4' | 0.31 | 1.51 | 0.16 | -0.19 | 0.49 |

(D) List of genes up-regulated in CD133 signature and down-regulated in at least one of the GBM subtypes with corresponding GBM/Normal fold changes.

|  | **Gene symbol** | **Mesenchymal** | **Classical** | **Neural** | **Proneural** | **All GBM samples** |
| --- | --- | --- | --- | --- | --- | --- |
| **1** | 'SST' | -6.64 | -6.56 | -5.33 | -6.09 | -6.23 |
| **2** | 'SNCA' | -4.36 | -4.85 | -3.71 | -4.46 | -4.38 |
| **3** | 'A2BP1' | -4.61 | -4.59 | -3.68 | -4.24 | -4.33 |
| **4** | 'NPY' | -4.75 | -4.90 | -3.10 | -3.89 | -4.27 |
| **5** | 'HS3ST2' | -4.35 | -4.58 | -3.66 | -4.25 | -4.25 |
| **6** | 'ERC2' | -4.11 | -5.14 | -3.55 | -4.00 | -4.24 |
| **7** | 'PHYHIP' | -4.40 | -4.45 | -3.52 | -4.15 | -4.18 |
| **8** | 'SCN2A' | -4.51 | -4.34 | -3.60 | -3.68 | -4.10 |
| **9** | 'LY6H' | -4.39 | -4.69 | -3.19 | -3.66 | -4.07 |
| **10** | 'TUBB4' | -4.65 | -5.14 | -2.76 | -2.86 | -4.01 |
| **11** | 'PDE2A' | -4.18 | -4.26 | -3.20 | -3.45 | -3.84 |
| **12** | 'MAL' | -3.75 | -4.54 | -2.39 | -3.68 | -3.67 |
| **13** | 'DIRAS2' | -4.23 | -3.27 | -2.77 | -3.41 | -3.50 |
| **14** | 'DLG2' | -3.66 | -3.67 | -2.97 | -3.42 | -3.47 |
| **15** | 'PPP1R16B' | -3.76 | -4.05 | -2.78 | -2.89 | -3.45 |
| **16** | 'CALB1' | -3.01 | -3.45 | -3.09 | -3.61 | -3.27 |
| **17** | 'RASL10A' | -3.54 | -3.33 | -1.96 | -2.86 | -3.02 |
| **18** | 'RPH3A' | -3.72 | -2.09 | -2.87 | -3.15 | -2.99 |
| **19** | 'RASAL1' | -2.96 | -2.94 | -2.58 | -2.88 | -2.86 |
| **20** | 'CAP2' | -2.93 | -2.72 | -2.43 | -2.74 | -2.73 |
| **21** | 'CENTA1' | -2.82 | -3.07 | -2.19 | -2.70 | -2.73 |
| **22** | 'RELN' | -2.89 | -3.03 | -1.88 | -2.61 | -2.66 |
| **23** | 'OMG' | -3.78 | -2.93 | -1.36 | -1.67 | -2.61 |
| **24** | 'WFDC1' | -2.74 | -2.66 | -2.14 | -2.48 | -2.54 |
| **25** | 'WFDC2' | -2.54 | -2.49 | -2.52 | -2.57 | -2.53 |
| **26** | 'DKFZP564O0823' | -2.36 | -2.87 | -2.16 | -2.65 | -2.52 |
| **27** | 'GLS2' | -2.59 | -2.58 | -2.17 | -2.53 | -2.49 |
| **28** | 'STAT4' | -2.30 | -2.56 | -2.15 | -2.63 | -2.41 |
| **29** | 'FGF9' | -2.87 | -2.81 | -1.91 | -1.49 | -2.36 |
| **30** | 'ACCN1' | -2.39 | -2.33 | -2.14 | -2.41 | -2.33 |
| **31** | 'PPM1H' | -2.53 | -2.45 | -1.90 | -2.16 | -2.30 |
| **32** | 'C10orf116' | -2.42 | -2.58 | -1.29 | -2.38 | -2.23 |
| **33** | 'TNNT1' | -2.10 | -2.33 | -2.05 | -2.02 | -2.13 |
| **34** | 'KLK7' | -2.12 | -2.15 | -2.03 | -2.14 | -2.11 |
| **35** | 'S100A1' | -2.31 | -2.71 | -1.12 | -1.74 | -2.05 |
| **36** | 'PVALB' | -2.08 | -2.08 | -1.59 | -2.07 | -1.98 |
| **37** | 'C14orf162' | -2.07 | -2.03 | -1.77 | -1.95 | -1.97 |
| **38** | 'PLLP' | -2.67 | -2.15 | -1.19 | -1.37 | -1.95 |
| **39** | 'ATP1A2' | -2.96 | -1.32 | -0.47 | -2.21 | -1.86 |
| **40** | 'PIGZ' | -1.98 | -1.75 | -1.66 | -1.75 | -1.81 |
| **41** | 'COBL' | -2.27 | -1.65 | -1.23 | -1.82 | -1.80 |
| **42** | 'B3GAT1' | -2.32 | -1.50 | -1.45 | -1.33 | -1.71 |
| **43** | 'TACSTD1' | -1.67 | -1.77 | -1.63 | -1.73 | -1.70 |
| **44** | 'MAP7' | -1.71 | -1.80 | -1.11 | -1.88 | -1.65 |
| **45** | 'NDEL1' | -1.39 | -1.56 | -1.59 | -1.69 | -1.54 |
| **46** | 'GPR88' | -1.68 | -1.69 | -0.86 | -1.77 | -1.53 |
| **47** | 'DHRS9' | -1.78 | -1.84 | -0.36 | -1.65 | -1.48 |
| **48** | 'REPS2' | -1.41 | -1.80 | -1.18 | -1.17 | -1.41 |
| **49** | 'ASRGL1' | -1.88 | -1.64 | -0.98 | -0.84 | -1.41 |
| **50** | 'DPP6' | -2.37 | -0.74 | -1.01 | -1.14 | -1.40 |
| **51** | 'STYK1' | -1.44 | -1.42 | -1.27 | -1.40 | -1.39 |
| **52** | 'PPP1R9A' | -1.70 | -1.63 | -1.04 | -0.95 | -1.38 |
| **53** | 'SCGB2A1' | -1.38 | -1.34 | -1.26 | -1.44 | -1.36 |
| **54** | 'OCA2' | -1.38 | -1.38 | -1.30 | -1.33 | -1.35 |
| **55** | 'FLJ20160' | -1.42 | -1.47 | -1.13 | -1.20 | -1.33 |
| **56** | 'CKB' | -2.26 | -0.68 | -0.90 | -1.08 | -1.31 |
| **57** | 'FAM107A' | -1.78 | -1.30 | -0.07 | -1.64 | -1.28 |
| **58** | 'SNX10' | -0.55 | -1.92 | -1.04 | -1.29 | -1.17 |
| **59** | 'CCDC28A' | -0.85 | -1.22 | -0.89 | -1.35 | -1.07 |
| **60** | 'SEMA3G' | -1.06 | -1.06 | -0.88 | -1.21 | -1.06 |
| **61** | 'PRODH' | -1.46 | -0.92 | -0.30 | -1.25 | -1.04 |
| **62** | 'HOOK1' | -1.05 | -1.07 | -0.93 | -0.97 | -1.01 |
| **63** | 'JAG2' | -1.00 | -1.05 | -0.97 | -1.00 | -1.01 |
| **64** | 'SEMA6B' | -0.99 | -1.04 | -0.95 | -0.91 | -0.98 |
| **65** | 'LCN2' | -0.87 | -0.91 | -0.94 | -1.02 | -0.93 |
| **66** | 'ADRBK2' | -0.90 | -1.08 | -0.74 | -0.93 | -0.92 |
| **67** | 'RNASE1' | -0.08 | -1.73 | -0.95 | -1.10 | -0.91 |
| **68** | 'KRT18' | -0.57 | -1.02 | -1.12 | -1.07 | -0.91 |
| **69** | 'CCL4' | -0.62 | -1.67 | -0.44 | -0.83 | -0.91 |
| **70** | 'CD83' | -0.62 | -1.28 | -0.74 | -0.98 | -0.90 |
| **71** | 'CUGBP2' | -0.98 | -1.04 | -0.72 | -0.57 | -0.85 |
| **72** | 'ENPP4' | -0.59 | -1.74 | -0.29 | -0.58 | -0.83 |
| **73** | 'NCAN' | -2.06 | -0.35 | -0.17 | -0.18 | -0.82 |
| **74** | 'GALNAC4S-6ST' | -0.42 | -1.19 | -0.47 | -1.11 | -0.79 |
| **75** | 'ALDH1A1' | -0.47 | -1.26 | 0.03 | -1.30 | -0.76 |
| **76** | 'PADI2' | -0.54 | -1.06 | -0.04 | -1.00 | -0.68 |
| **77** | 'AKR1C3' | -0.61 | -1.47 | 0.41 | -0.74 | -0.66 |
| **78** | 'RYR3' | -0.75 | 0.23 | -0.50 | -1.72 | -0.66 |
| **79** | 'DEPDC6' | -0.37 | -1.17 | 0.17 | -0.86 | -0.58 |
| **80** | 'SCN3A' | -1.45 | -1.12 | -0.04 | 0.93 | -0.56 |
| **81** | 'ABAT' | -1.20 | -0.26 | -0.03 | -0.05 | -0.47 |
| **82** | 'NR2E1' | -0.24 | 0.64 | -0.33 | -1.63 | -0.33 |
| **83** | 'P2RY13' | -0.07 | -0.62 | 0.54 | -1.03 | -0.30 |
